# Development of the dictyostelid *Polysphondylium violaceum* does not require secreted cAMP

**DOI:** 10.1242/bio.059728

**Published:** 2023-02-02

**Authors:** Yoshinori Kawabe, Pauline Schaap

**Affiliations:** School of Life Sciences, Molecular, Cell and Developmental Biology, University of Dundee, Dundee DD15EH, UK

**Keywords:** Evolution of cell-cell communication, Clade-specific invention, Cell-type specialization, CAMP oscillations, Morphogenetic movement, Chemotaxis

## Abstract

Group 4 Dictyostelia, like *Dictyostelium discoideum*, self-organize into aggregates and fruiting bodies using propagating waves of the chemoattractant cAMP, which are produced by a network containing the adenylate cyclase AcaA, cAMP receptors (Cars) and the extracellular cAMP phosphodiesterase PdsA. Additionally, AcaA and the adenylate cyclases AcrA and AcgA produce secreted cAMP for induction of aggregative and prespore gene expression and intracellular cAMP for PKA activation, with PKA triggering initiation of development and spore and stalk maturation. Non-group 4 species also use secreted cAMP to coordinate post-aggregative morphogenesis and prespore induction but use other attractants to aggregate. To understand how cAMP's role in aggregation evolved, we deleted the *acaA*, *carA* and *pdsA* genes of *Polysphondylium violaceum*, a sister species to group 4. *acaAˉ* fruiting bodies had thinner stalks but otherwise developed normally. Deletion of *acrA*, which was similarly expressed as *acaA*, reduced aggregation centre initiation and, as also occurred after *D. discoideum acrA* deletion, caused spore instability. Double *acaAˉacrAˉ* mutants failed to form stable aggregates, a defect that was overcome by exposure to the PKA agonist 8Br-cAMP, and therefore likely due to reduced intracellular cAMP. The *carAˉ* and *pdsAˉ* mutants showed normal aggregation and fruiting body development. Together, the data showed that *P. violaceum* development does not critically require secreted cAMP, while roles of intracellular cAMP in initiation of development and spore maturation are conserved. Apparently, cell-cell communication underwent major taxon-group specific innovation in Dictyostelia.

## Teaser

**Summary:** Despite evidence that secreted cAMP is widely used by most Dictyostelia to control morphogenesis and cell differentiation, *Polysphondylium violaceum* does not require genes responsible for detection, synthesis and degradation of secreted cAMP

## INTRODUCTION

Multicellularity evolved independently in seven of the eight major divisions of eukaryotes (Brown et al., 2012; Du et al., 2015; Tice et al., 2016) and in prokaryotes (Lyons and Kolter, 2015). Multicellular forms range from simple clumps or mats of cells to a myriad of complex organisms composed of many specialized cells, arranged in tissues and organs. The regulation of cell-type specialization and the proper positioning of the specialized cells within the organism requires extensive cell to cell communication mechanisms.

Comparative genomics has highlighted that many proteins involved in mediating and processing intercellular communication are deeply conserved throughout multicellular lineages (Gold et al., 2019; Srivastava et al., 2010), while those involved in intracellular processing can often be retraced to their unicellular ancestors (King et al., 2008; Suga et al., 2013). Nevertheless, there are only few narratives that connect signalling mechanisms in a unicellular ancestor with developmental control in a multicellular descendant. One example is the co-option of a transcription factor, RSL1, that mediates stress response in the unicellular alga *Chlamydomonas reinhardtii* into a developmental role as RegA, inducing somatic cell fate in its multicellular relative *Volvox carteri* (König and Nedelcu, 2020). Another example is the evolution of cell-type specialization and morphogenesis in the Dictyostelia from an ancestral stress response.

Dictyostelia are members of Amoebozoa, a eukaryote division that mostly consists of single-celled amoebas, which encapsulate to form walled cysts when starved. Instead, starving Dictyostelia aggregate to form multicellular fruiting bodies that consists of walled spores and stalk cells. The model species *D. discoideum* (*Ddis*), uses cAMP as attractant to coordinate aggregation and fruiting body morphogenesis. For this role, cAMP is secreted in pulses, which are generated by a network that contains the adenylate cyclase AcaA (Pitt et al., 1992), G-protein coupled cAMP receptors (Cars) (Sun et al., 1990), an extracellular phosphodiesterase, PdsA (Sucgang et al., 1997), and some intracellular components. Secreted cAMP also regulates developmental gene expression and induces the expression of prespore genes. In addition, cAMP acts an intracellular messenger for external signals that trigger the growth to development transition and the encapsulation of spores and stalk cells. Here, cAMP activates cAMP-dependent protein kinase (PKA) and is mostly synthesized by the adenylate cyclases AcgA and AcrA and hydrolysed by the intracellular phosphodiesterase RegA (Du et al., 2015; Loomis, 2014).

Molecular phylogeny partitions Dictyostelia into four major and some minor groups (Schaap et al., 2006; Schilde et al., 2019). Many species in groups 1, 2 and 3 can still individually encyst in addition to forming fruiting bodies, but group 4, which contains *Ddis*, has lost encystation (Romeralo et al., 2013). Comparative studies revealed that the roles of PKA, AcgA, AcrA and RegA in *Ddis* spore and stalk encapsulation are evolutionary derived from roles as intermediates for stress-induced encystation in both Dictyostelia and single-celled Amoebozoa (Du et al., 2014; Kawabe et al., 2015; Ritchie et al., 2008).

Group 4 species all use cAMP as the attractant for aggregation, but most species in groups 1, 2 and 3 appear to use the dipeptide glorin (Romeralo et al., 2013), that was initially identified as the attractant of *Polysphondylium violaceum* (*Pvio*) (Shimomura et al., 1982). Deletion of *pdsA* and the *carA* and *carB* genes of the group 2 species *Polysphondylium pallidum* (*Ppal*) did not affect aggregation (as expected) but disrupted fruiting body morphogenesis. The *carAˉcarBˉ* cells also lost prespore differentiation and differentiated as cysts in the aberrant fruiting structures (Kawabe et al., 2009, 2012). These data indicated that secreted cAMP coordinated post-aggregative morphogenesis and induced prespore differentiation in *Ppal*, as it does in *Ddis*, and supported a hypothesis whereby first intracellular and next extracellular cAMP signalling in Dictyostelia evolved from cAMP-mediated encystation in the unicellular ancestor (Kin and Schaap, 2021).

We recently developed gene knock-out procedures and generated genome and transcriptome sequence data for *Pvio*, a species that resides in a small sister group to group 4 (Narita et al., 2020). This position and the fact that it only has single copies of the *acaA*, *car* and *pdsA* genes makes *Pvio* uniquely suited to investigate how secreted cAMP signalling evolved to its very dominant role in group 4. The gene disruption studies led to the surprising finding that in *Pvio* development extracellular cAMP signalling plays no role of significance at all.

## RESULTS

### Investigation of AcaA function in *P. violaceum*

*Pvio* is one of the earliest identified species of Dictyostelia (Brefeld, 1884) and is characterized by aggregates with inflowing streams, the formation of whorls of side branches from cell masses that pinch off from the primary sorogen and the violet colour of its mature spores. It is the founding species of the genus *Polysphondylium*, which all share the whorled fruiting bodies, but not the violet colour. Classification based on molecular data later showed that the whorled phenotype was polyphyletic (Schaap et al., 2006; Schilde et al., 2019) and that white polyphondylids were members of group 2 of the 4 major dictyostelid taxon groups, while *Pvio* was part of a small sister clade to group 4, with the latter containing *Ddis* and other species that use cAMP as attractant. The *Pvio* attractant was identified as a dipeptide of glutamate and ornithine, called glorin (Shimomura et al., 1982) and appears to be widely used among non-group 4 species (Romeralo et al., 2013). Genome and transcriptome data for *Pvio* Qsvi11 are available (Narita et al., 2020) and this strain can be transformed with vectors harbouring G418 or hygromycin selection cassettes. Gene knock-out by homologous transformation proved thus far to be very efficient, usually occurring in 80-100% of transformed clones (Narita et al., 2020). The whorled phenotype of Qsvi11 is, however, variable and whorls are often sparse, making it difficult to evaluate effects of gene manipulation on whorl formation.

**Fig. 1.**
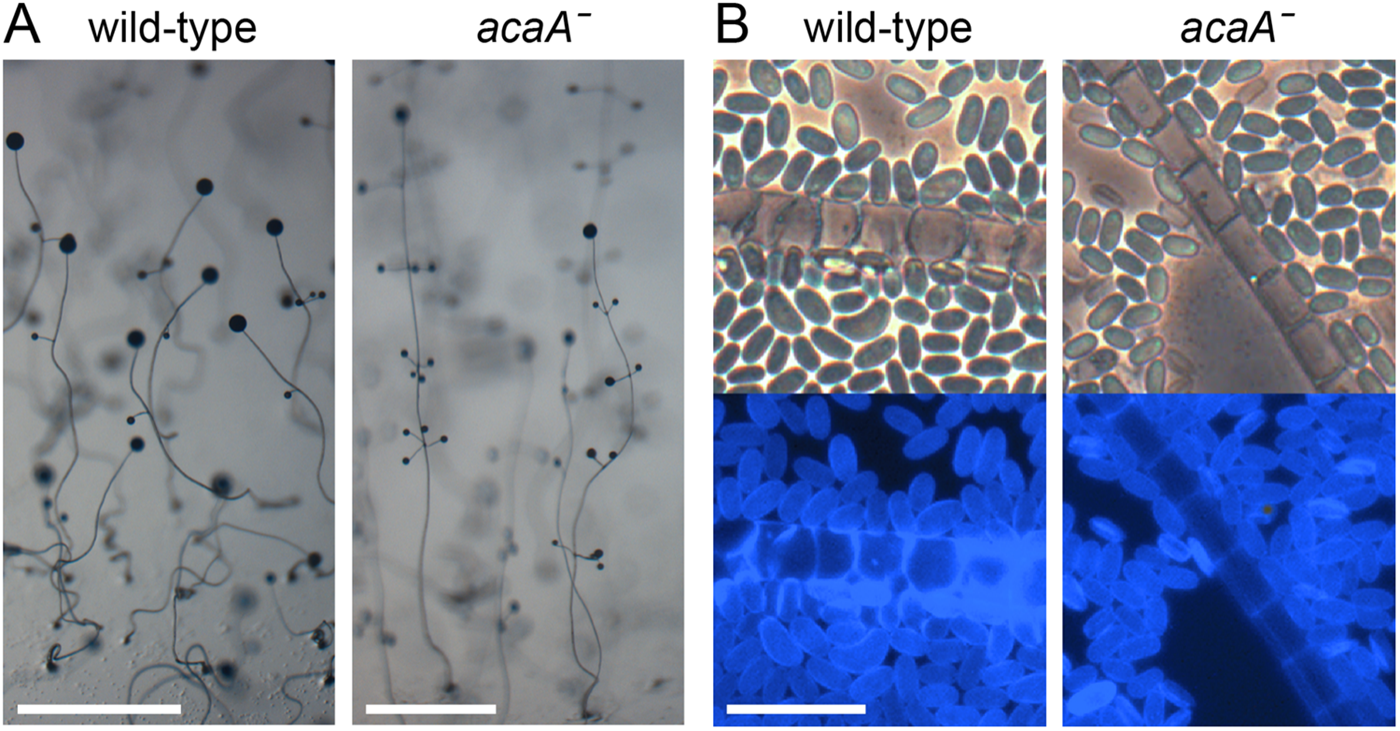
**Development and differentiation of the *Pvio acaA*ˉ mutants.** *Pvio* wild-type and *acaAˉ* cells were incubated overnight at 4°C on KK2 agar at 10^6^ cells/cm^2^ and then at 22°C until mature fruiting bodies had formed. (A) Fruiting body morphology of wild type and *acaAˉ* cells. Scale bars: 1.0 mm. (B) Mature fruiting structures were stained with 0.001% Calcofluor *in situ* and photographed under phase contrast (top) and epifluorescence (bottom). Scale bar: 20 µm.

*AcaA* is encoded by a single copy gene in *Pvio* (Fig. S1) and to assess its role in post-aggregative morphogenesis, we deleted *acaA* by homologous recombination (Fig. S2A). The *acaAˉ* clones aggregated normally and formed fruiting bodies with somewhat thinner and longer stalks than those of wild-type cells (Fig. 1A). This was also evident in Calcofluor stained structures at higher magnification, where the length/width ratio of *acaAˉ* stalk cells was greater than that of wild-type stalk cells (Fig. 1B). The *acaAˉ* mutant formed normal elliptical spores.

### Phenotypes of *Pvio acrAˉ* and *acaAˉacrAˉ* double mutants

Similar to other Dictyostelia, *Pvio* has two other adenylate cyclase genes, *acrA* and *acgA* (Fig. S1). *Ddis acrAˉ* cells form fruiting bodies with thin stalks and spores that germinate precociously in the spore head (Soderbom et al., 1999), while *acgAˉ* mutants show only sporulation defects (Alvarez-Curto et al., 2007; Van Es et al., 1996). *Pvio acgA* is predominantly expressed in spores in late development (Fig. S1), while *acrA* is like *acaA* upregulated during aggregation and stalk-enriched (Fig. S1). To examine the expression pattern of both genes in developing structures, we transformed wild-type *Pvio* with fusion constructs of the *acaA* or *acrA* promoters and the *lacZ* reporter. Expression of β-galactosidase from either promoter was enriched in aggregation centres and was later present throughout the primary and secondary sorogens and the stalk (Fig. S3). Expression from the *acaA* promoter was somewhat stronger at the sorogen tips. Because the expression pattern of *acrA* is almost the same as that of *acaA*, we investigated whether AcrA might compensate for loss of AcaA in *Pvio* by disrupting *acrA* in wild-type and *acaAˉ* cells (Fig. S2B).

**Fig. 2.**
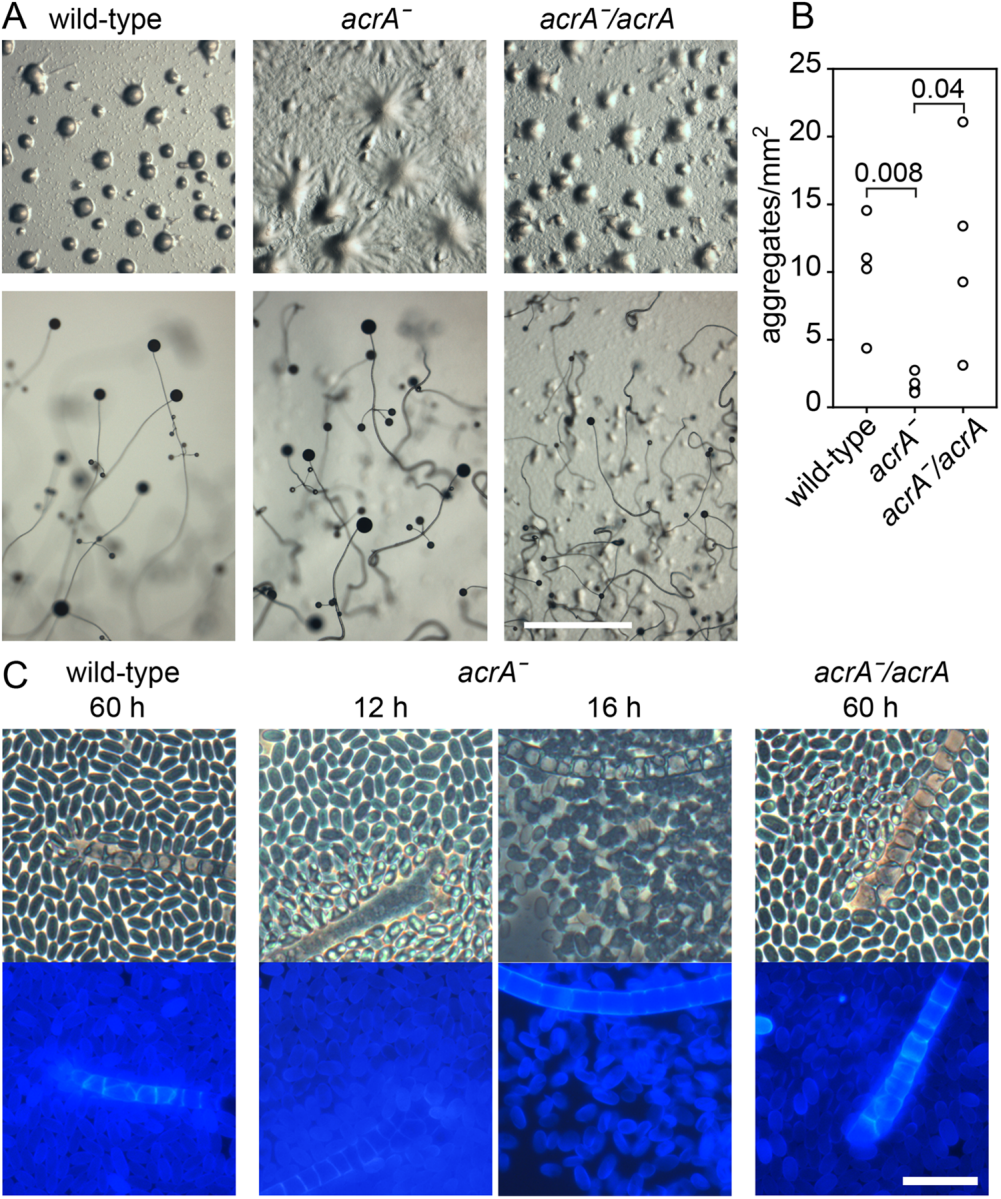
**Phenotype of *Pvio acrA*ˉ and *acrA*ˉ/*AcrA* mutants.** (A) Development and fruiting body morphology. *Pvio* wild-type, *acrAˉ* and *acrAˉ* cells, complemented with *acrA* expressed from its own promoter, were plated on KK2 agar at 10^6^ cells/cm^2^ and incubated until cells had formed aggregates (top) and mature fruiting bodies (bottom). Scale bar: 1 mm. (B) Aggregate density. After aggregation was completed, the number of aggregates per mm^2^ was determined. Data of four independent experiments, measuring 108 to 322 aggregates for per cell line, each, are shown with *P*-values of a *t*-test. The data passed normality and equal variance tests. (C) Sporulation. Cells were starved overnight at 4°C and then for 12 h to 60 h at 22°C. Mature fruiting structures were stained with 0.001% Calcofluor *in situ* and photographed under phase contrast (top) and epifluorescence (bottom). Scale bar: 20 µm.

The *Pvio acrA* knock-out clones formed large aggregates and robust fruiting bodies (Fig. 2A). Measurement of aggregate density showed that *acrAˉ* cells initiated about eight times less aggregates/mm^2^ than wild-type cells (Fig. 2B), which is the likely cause of the increased size of their aggregates and fruiting bodies. Aggregation of *acrAˉ* cells also seemed a bit delayed, but because the timing of aggregation is also variable in wild-type *Pvio*, we cannot state this with certainty. Calcofluor staining revealed that *acrAˉ* cells initially formed normal spores and stalk cells in early fruiting bodies (Fig. 2C, 12 h), but that 4 h later the spore head contained many amoebas and empty spore walls, indicating that spores had precociously germinated. Precocious spore germination is also reported for *Ddis acrAˉ* (Soderbom et al., 1999). Expression of *acrA* in *acrAˉ* cells restored both the reduced aggregate density (Fig. 2A) and precocious germination defects of *acrAˉ* (Fig. 2C), indicating that these phenotypes were caused by *acrA* gene disruption.

**Fig. 3.**
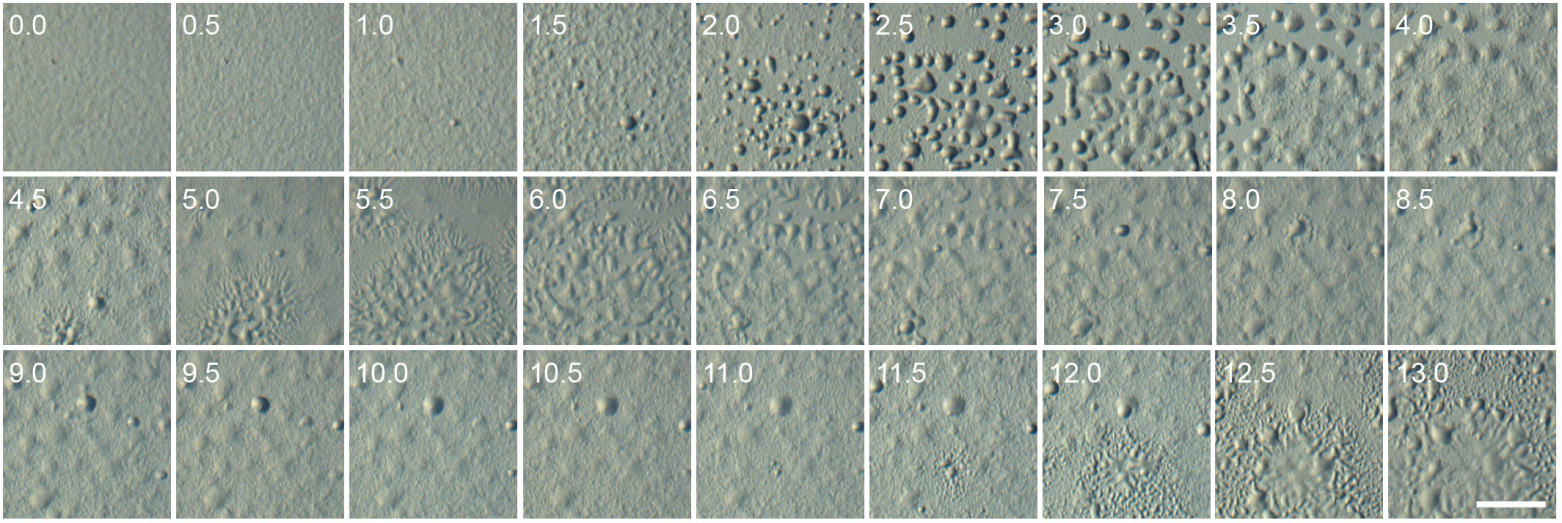
**Development of a *Pvio acaA*ˉ*acrA*ˉ mutant.** *Pvio acaA*ˉ*acrA*ˉ cells were incubated overnight at 4°C on KK2 agar at 10^6^ cells/cm^2^ and then transferred to 22°C. The numbers on the upper left indicate hours of incubation at 22°C. Scale bar: 0.5 mm.

**Fig. 4.**
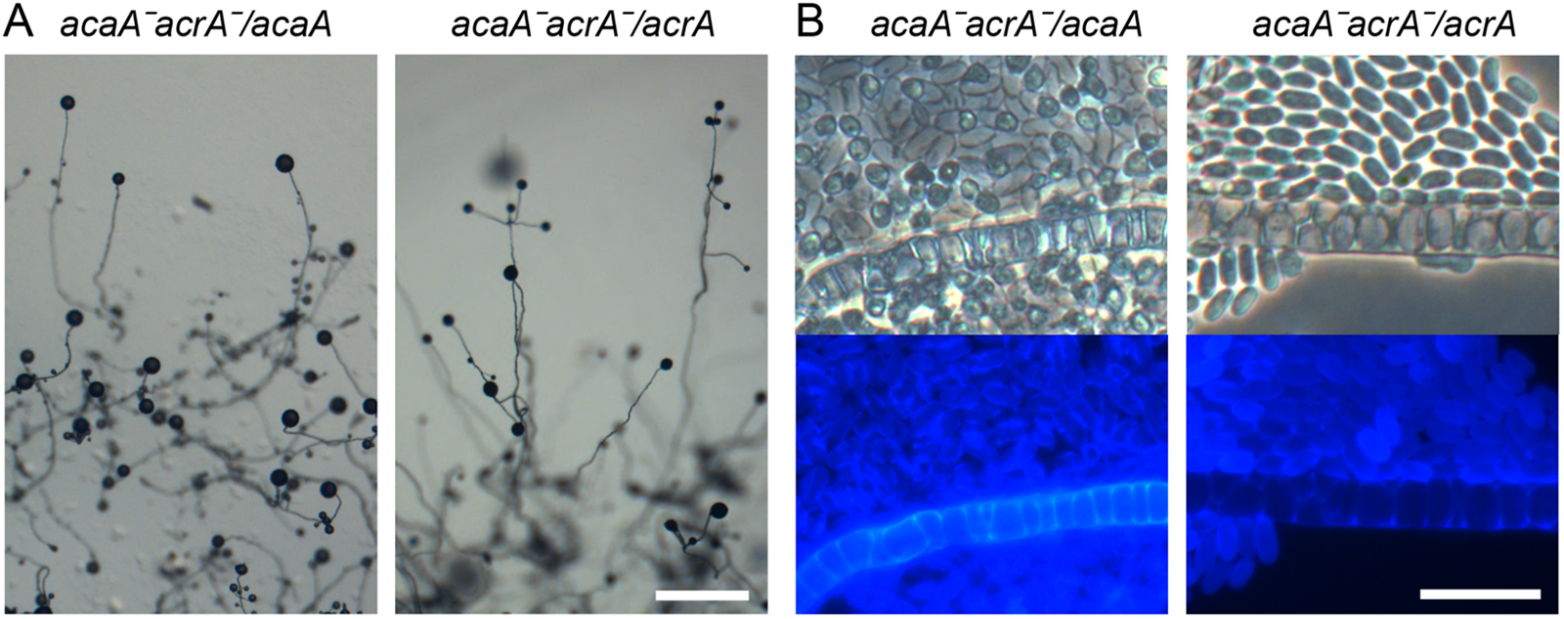
**Complementation of *acaAˉacrAˉ* with *acaA* or *acrA.*** (A) Fruiting body morphology. The *acaA*ˉ*acrA*ˉ mutant was transformed with *acaA* or *acrA* expressed from their own promoter. The transformants were developed into fruiting bodies and photographed. Scale bar: 1 mm. (B) Spore and stalk cells. Mature fruiting structures were stained with 0.001% Calcofluor *in situ* and photographed under phase contrast (top) and epifluorescence (bottom). Scale bar: 20 µm.

The *Pvio acaAˉacrAˉ* mutant was severely defective. It initially aggregated upon starvation but never formed sorogens (Fig. 3). Instead, the aggregated cells dispersed within a few hours and then formed aggregates again. This process was repeated several times at about 5-7 h intervals during at least 24 h. Normal development was restored by expression of either *acaA* or *acrA* from their own promoters (Fig. 4). Each of the complemented strains formed fruiting bodies, but the spores of *acaAˉacrAˉ* transformed with *acaA* germinated in spore head (Fig. 4B), as was the case for *acrAˉ* mutants. The restoration of fruiting body formation indicates that *acaA* and *acrA* have overlapping roles in stabilizing aggregates to initiate progression into fruiting body formation.

### Restoration of *acaAˉacrAˉ* development by 8Br-cAMP

**Fig. 5.**
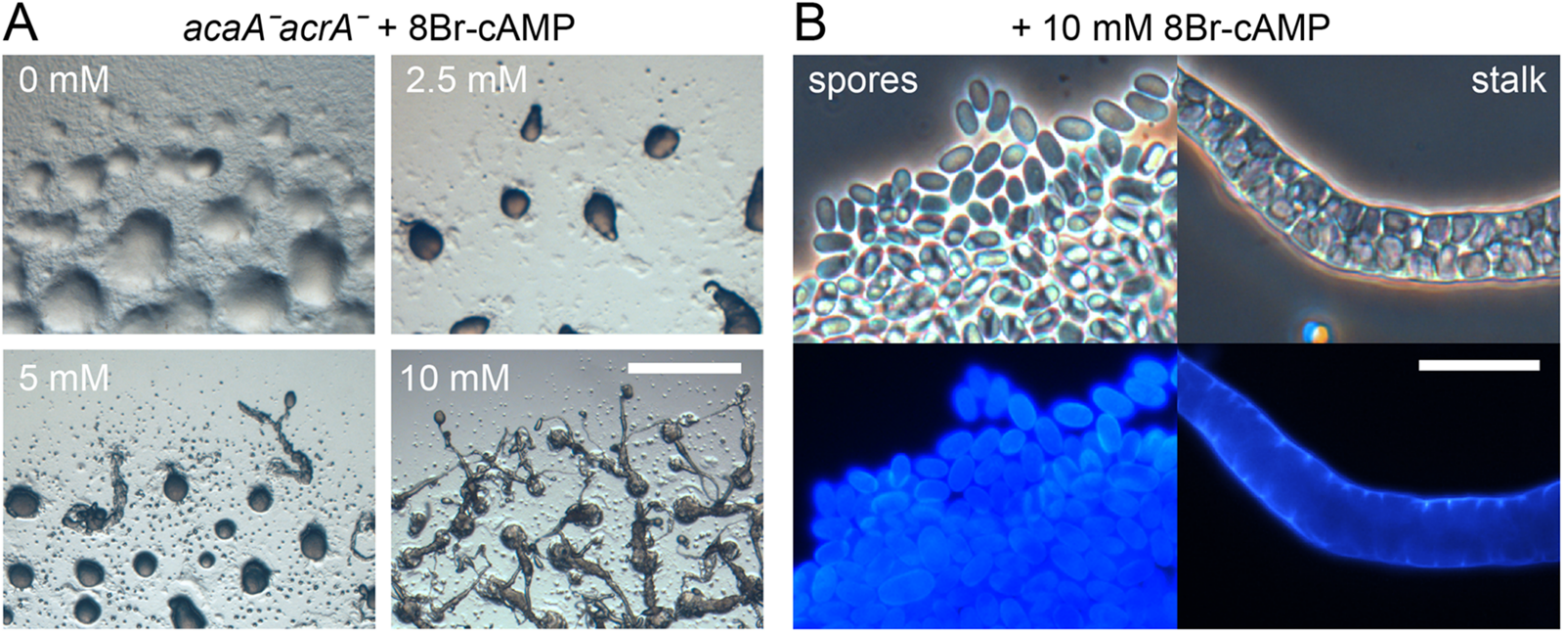
**Induction of fruiting body formation in *acaA*ˉ*acrA*ˉ with 8Br-cAMP.** (A) Development. *acaA*ˉ*acrA*ˉ cells were incubated for 24 h on KK2 agar containing the indicated concentrations of 8Br-cAMP and photographed *in situ.* Scale bar: 1 mm. (B) Spore and stalk cells. The *acaA*ˉ*acrA*ˉ structures, developed on agar containing 10 mM 8Br-cAMP, were stained with 0.001% Calcofluor and photographed under phase contrast (top) and epifluorescence (bottom). Scale bar: 20 µm.

In *Ddis*, fruiting body formation requires both extracellular cAMP acting on Cars for organisation of morphogenesis and induction of prespore gene expression (Singer et al., 2019; Wang et al., 1988) and intracellular cAMP acting on PKA for induction of spore and stalk cell maturation (Harwood et al., 1992; Hopper et al., 1993). To address which cAMP signalling defect caused the *acaAˉ*/*acrAˉ* phenotype, *Pvio acaAˉ*/*acrAˉ* cells were developed on agar containing 8Br-cAMP, a membrane-permeant cAMP analogue with high affinity for PKA, but not Cars (Van Haastert and Kien, 1983). At 2.5 mM 8Br-cAMP, stable tipped aggregates were formed that did not develop further, but at 5 mM and more so at 10 mM 8Br-cAMP, the tipped mounds developed into stalked structures with spore heads, which, though morphologically aberrant, showed normal elliptical spores and vacuolated stalk cells (Fig. 5). These results show that direct activation of PKA compensates fully for defective aggregation, sporulation and stalk cell differentiation in the *acaAˉacrAˉ* and partially for defective morphogenesis.

### Effects of deletion of *Pvio carA* and *PdsA* and Sp-cAMPS

The partial restoration of *Pvio acaAˉacrAˉ* development by 8Br-cAMP indicates that AcaA and AcrA have a shared role in providing intracellular cAMP for PKA activation. However, this does not exclude that secreted cAMP pulses are not important for *Pvio* development as well. In *Ddis*, both the cAMP receptor CarA and the extracellular cAMP phosphodiesterase PdsA are essential for generating cAMP pulses, while CarA also mediates cAMP induction of chemotaxis and prespore gene expression (Schaap and Van Driel, 1985; Sucgang et al., 1997; Sun et al., 1990). BlastP searches revealed that *Pvio* has only a single *pdsA* and a single *carA* gene, which were respectively amplified once and three times in group 4 (Fig. S4).

**Fig. 6.**
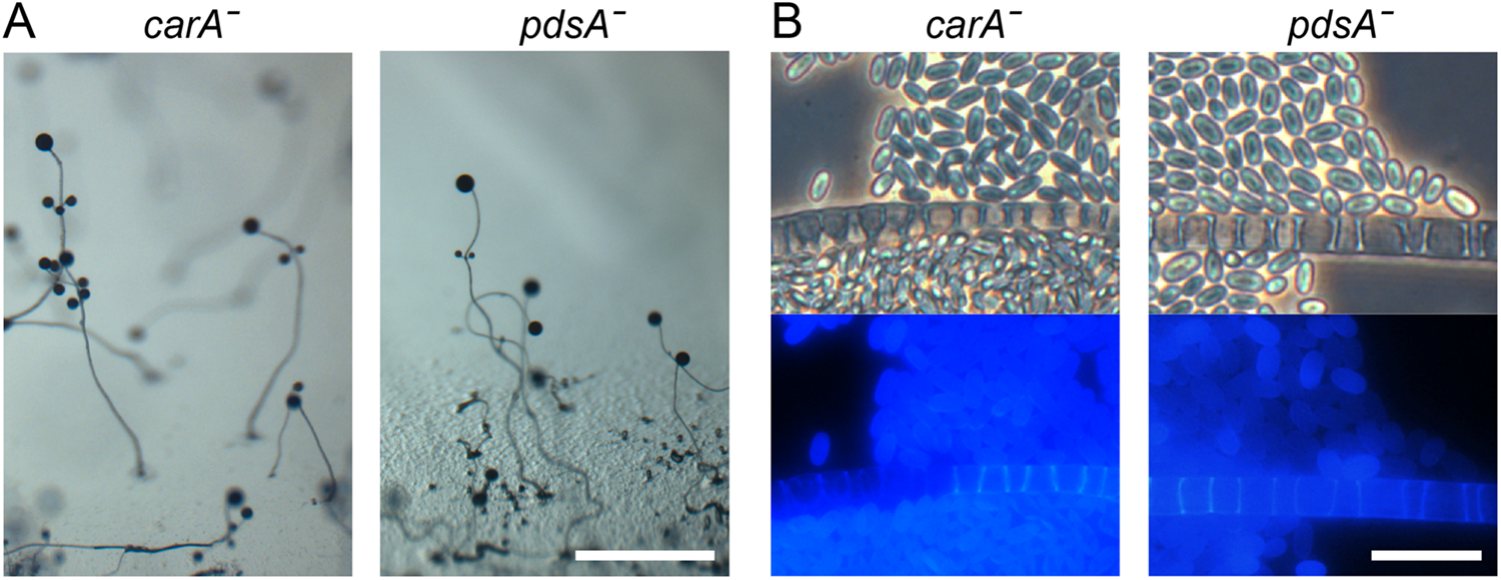
**Development and differentiation of *carA*ˉ and *pdsA*ˉ *Pvio* cells.** (A) Fruiting body morphology. Scale bar: 1 mm. (B) Spore and stalk cells*.* Mature fruiting structures were stained with 0.001% Calcofluor *in situ* and photographed under phase contrast (top) and epifluorescence (bottom). Scale bar: 20 µm.

We deleted the single *carA* and *pdsA* genes to evaluate roles for secreted cAMP in *Pvio* (Fig. S5). Both the *carAˉ* and *pdsAˉ* mutants aggregated normally and constructed fruiting bodies (Fig. 6A). Calcofluor staining revealed that these fruiting bodies were composed of mature spores and stalk cells whose morphologies were also normal. These results suggest that secreted cAMP does not play a significant role in *Pvio* aggregation, fruiting body development or cell differentiation. To further test involvement of oscillatory cAMP signalling in *Pvio*, we developed cells on agar containing 10, 50 or 100 μM on the slowly hydrolysable cAMP analogue Sp-cAMPS, which inhibits cAMP responses like chemotaxis and cAMP relay that are subject to adaptation, and thereby inhibit *Ddis* development, starting from 0.5 μM (Rossier et al., 1978). Fig. S6 shows that up to 100 μM Sp-cAMPS had no effect on *Pvio* aggregation and development into fruiting bodies, consolidating the evidence that *Pvio* does not require dynamic extracellular cAMP signalling.

## DISCUSSION

### AcaA does not critically regulate *P. violaceum* development

Phylogenetic comparative methods showed that major innovations occurred in the last common ancestor (LCA) to the group 4 dictyostelids, such as the formation of robust solitary unbranched fruiting bodies with two novel somatic cell types, the early fate mapping of spore and stalk cells and extensive slug migration. Species in groups 1, 2 and 3 form smaller grouped or branched fruiting bodies with stalk cells as the only somatic cell type, and the stalk is formed by local redifferentiation of prespore cells. The changes in group 4 are accompanied by the loss of encystation as an alternative survival strategy and the use of secreted cAMP pulses to coordinate chemotactic aggregation (Romeralo et al., 2013; Schilde et al., 2014).

Deletion of *car* and *pdsA* genes in the group 2 species *Ppal* indicated that non-group 4 species still used secreted cAMP to organize post-aggregative morphogenesis and prespore differentiation (Alvarez-Curto et al., 2005; Kawabe et al., 2002, 2009, 2012). This notion was supported by findings that the group 3 species *D. minutum*, displayed cAMP stimulated cAMP synthesis (Schaap, 1985) and optical density waves after aggregation, with the latter being disrupted by the non-hydrolysable cAMP analogue Sp-cAMPS (Schaap et al., 1984). Sp-cAMPS also disrupted post-aggregative morphogenesis of most other non-group 4 species (Romeralo et al., 2013).

With its position in the closest sister clade to group 4, *Pvio* is well placed for investigating the molecular changes that occurred in the LCA to group 4 that caused the dramatic innovations in this group. An added advantage is the efficiency of gene knock-out in *Pvio* (Narita et al., 2020) and that its attractant, glorin, is known (Shimomura et al., 1982). To particularly understand the evolution of secreted cAMP signalling, we deleted the single gene encoding *AcaA*, the pivotal enzyme in the network that generates the secreted cAMP pulses that control *Ddis* aggregation and post-aggregative morphogenesis (Patel et al., 2000; Pitt et al., 1992; Singer et al., 2019).

The loss of AcaA function in *Pvio* did not markedly affect aggregation, fruiting body morphogenesis or spore and stalk cell differentiation. Only stalks appeared somewhat thinner with the individual stalk cells showing a lower width to length ratio (Fig. 1). This suggested that either secreted cAMP played no mayor role in *Pvio* or the role of AcaA was shared with another adenylate cyclase.

### *P. violaceum* AcaA and AcrA are together required for aggregation

Apart from a sporulation defect, *Ddis acrAˉ* mutants also make long thin stalks (Soderbom et al., 1999), while *Pvio acrA* showed similar developmental regulation and cell type specificity as *acaA* (Figs S1 and S3*)*, suggesting that AcrA and AcaA functions may overlap. A *Pvio acrAˉ* mutant showed the same sporulation defect as *Ddis acrAˉ*, but its fruiting bodies were actually more robust than those of wild type (Fig. 2). This was due to the formation of less closely spaced and therefore larger aggregates, suggesting a requirement for AcrA in aggregation centre initiation. A *Pvio acaAˉacrAˉ* double mutant showed a more severe phenotype. It formed aggregates that never progressed into sorogens but disaggregated instead, only to repeat a cycle of aggregation and disaggregation several times over (Fig. 3). Sorogen and fruiting body formation of *Pvio acaAˉacrAˉ* was partially restored by developing mutants with the PKA agonist 8Br-cAMP (Fig. 5). While fruiting structures were morphologically aberrant, normal spores and vacuolated stalk cells were formed. *Pvio acaAˉacrAˉ* fruiting body morphogenesis was also restored by overexpression of either *acaA* or *acrA* (Fig. 4)*.* Since coordination of morphogenetic cell movement is attributed to the propagating cAMP waves that are produced by AcaA, this raised the question, whether cAMP waves are not required for *Pvio* morphogenesis.

### *P. violaceum* development does not require secreted cAMP

To address the question whether cAMP pulses or just secreted cAMP play any role in *Pvio* development, we deleted *pdsA*, which is essential for pulsatile cAMP signalling in *Ddis* (Sucgang et al., 1997) and *carA*, the only cAMP receptor gene that we could detect in the separately assembled genome and transcriptome of *Pvio* (Fig. S7). Both the *carAˉ* and the *pdsAˉ* mutants aggregated normally and formed normal fruiting bodies with mature spore and stalk cells. Unless other unrelated proteins have taken over CarA and PdsA function, this suggests that unlike *Ddis* and *Ppal*, secreted cAMP has no major role in coordination of cell movement or in the induction of prespore differentiation in *Pvio*. This notion was substantiated by *Pvio* aggregation and development proceeding normally in the presence of Sp-cAMPS (Fig. S6). This is a striking result considering that *Pvio* is more closely related to *Ddis*, with its multiple roles for secreted cAMP, than *Ppal*, where deletion of its two *car* genes or *pdsA* gene disrupts fruiting body morphogenesis and of *car* genes also spore differentiation (Kawabe et al., 2002, 2009, 2012). Evidently, not only group 4 but also *Pvio* underwent significant innovations in developmental control.

Which secreted signal(s) might have taken over the roles of secreted cAMP in *Pvio*? Here the *Pvio* chemoattractant glorin comes first to mind, since it was also reported to trigger gene expression in early *Ppal* development (Asghar et al., 2011). However, Dictyostelids are known to synthesize a range of other secondary metabolites that affect cell differentiation (Araki and Saito, 2019; Kikuchi et al., 2013; Kondo et al., 2019; Saito et al., 2022; Sasaki et al., 2020; Tsujioka et al., 2004) and focusing on one or a few compounds is therefore premature.

### Taxon group-specific functionalization of the Dictyostelid adenylate cyclases

Comparative analysis of adenylate cyclase function across Dictyostelia highlight considerable refunctionalisation of AcaA, AcrA and AcgA in the course of dictyostelid evolution. AcaA is essential in *Ddis* for producing the secreted cAMP pulses that organize aggregation and post-aggregative morphogenesis (Patel et al., 2000; Pitt et al., 1992), although a *Ddis acrAˉacgAˉacaAˉ* mutant that overexpresses PKA can still form mounds at high cell density (Hirose et al., 2021). *Ddis* AcaA also acts intracellularly in response to the stalk-inducer c-di-GMP to activate PKA and thereby stalk maturation (Chen and Schaap, 2012; Chen et al., 2017). However, apart from a reduction in stalk thickness, *Pvio acaAˉ* mutants showed no developmental abnormalities. *Ppal aca1ˉ* cells also form fruiting bodies with thinner stalks. However, even knock-outs in all three *Ppal aca* genes still form some fruiting bodies after a long delay (Kawabe and Schaap, 2022).

AcrA is required for robust stalk formation in *Ddis* and has an overlapping role with AcgA in induction of prespore differentiation and spore maturation (Alvarez-Curto et al., 2007; Soderbom et al., 1999). AcgA on its own mediates inhibition of spore germination by high osmolarity in the *Ddis* spore head (Van Es et al., 1996). However, neither AcrA nor AcgA or both together are essential for *Ppal* prespore and spore differentiation. They do have overlapping roles in mediating stress-induced encystation in *Ppal* and inhibition of spore and cyst germination by high osmolarity (Kawabe et al., 2015). Induction of prespore differentiation and spore maturation is dependent on both Cars and PKA in *Ppal* (Funamoto et al., 2003; Kawabe et al., 2009, 2015) as it is in *Ddis* (Hopper et al., 1993; Schaap and Van Driel, 1985). We surmised that in the *Ppal acrAˉacgAˉ* cells, any or all of the three *Ppal Aca* enzymes may provide cAMP for spore differentiation.

Similar to *Ddis* AcrA (Soderbom et al., 1999), *Pvio* AcrA is required for the differentiation of stable spores (Soderbom et al., 1999) (Fig. 2). *Pvio AcrA* is additionally required for efficient aggregation centre initiation and has an overlapping role with *AcaA* in the formation of stable aggregates. For these roles both AcaA and AcrA act upstream of PKA, since stable aggregation and fruiting body formation are restored in *acaAˉacrAˉ* mutants by 8Br-cAMP (Fig. 5). The distinction in *Ddis* between roles of AcaA in mostly extracellular Car activation, and roles of AcrA and AcgA in mostly PKA activation is therefore blurred in *Ppal* and *Pvio*.

Compared to other Amoebozoa, such as *Physarum polycephalum*, *Protostelium fungivorum* and *Acanthamoeba castellani* with, respectively, 64, 52 and 67 adenylate cyclases each (Clarke et al., 2013; Hillmann et al., 2018; Schaap et al., 2015), the number of adenylate cyclases in Dictyostelia is low. It is possible that in the unicellular ancestor of Dictyostelia the roles of their three adenylate cyclases were not highly specialized, i.e. they all responded to environmental stressors to induce the transition of amoebas into dormant cysts. In the newly emerging Dictyostelid taxon groups, the enzymes and their regulation may then have evolved independently to take on group-specific developmental roles.

## MATERIALS AND METHODS

### Growth and development

*P. violaceum* QSvi11, (Pvio) (Kalla et al., 2011), gift from J. E. Strassmann (Washington University in St. Louis, USA) was routinely grown in KK2 (16 mM KH_2_PO_4_ and 4 mM K_2_HPO_4_), containing autoclaved *Klebsiella aerogenes* (*K.aer*) (final OD_600_=8.5) and 10% HL5 shaken at 150 rpm. For some experiments cells were grown in association with *Escherichia coli* on 1/5^th^ SM agar (Formedium, UK). For multicellular development, cells were harvested from growth media and spread at 10^6^ cells/cm^2^ on KK2 agar (1.5% agar in KK2), incubated at 4°C overnight and then at 22°C until the desired developmental stage had been reached.

### DNA constructs and transformation

#### *AcaA* and *acrA* promoter-*lacZ* constructs and analysis

To construct a gene fusion of the promoter of *Pvio acaA* (Pvio_g2213*,* NCBI id.: KAF2076473) and *lacZ*, a fragment comprising the full 1.8 kb *acaA* 5'intergenic region and 0.1 kb 5′ coding sequence was amplified from *Pvio* gDNA, using primer pair Pv-ACA-P51 K and Pv-ACA-P31B (Table S1) that harbour *Kpn*I and *Bam*HI sites, respectively. After *Kpn*I/*Bam*HI digestion, the fragment was ligated into *Kpn*I/*Bam*HI digested pDdGal16 (Harwood and Drury, 1990), yielding vector pPv-acaA-LacZ.

To construct an *acrA_LacZ* fusion, a fragment from −822 to +125 nt relative to the start ATG of *acrA* (Pvio_g1249, KAF2077476) and containing the full 5′ intergenic region was amplified from *Pvio* gDNA, using primers Pv-ACB-P51X and Pv-ACB-P31B (Table S1) that harbour *Xba*I and *Bam*HI sites, respectively. After digestion the fragment was ligated into *Xba*I/*Bam*HI digested pDdGal16, yielding vector pPv-acrA-LacZ.

Both plasmids were validated by DNA sequencing and transformed into *Pvio* cells by electroporation. Transformants were selected at 50 µg/ml G418 on growth plates with G418 resistant *E. coli* (Narita et al., 2020). Transformed cells were developed on dialysis membrane supported by KK2 agar and β-galactosidase activity was visualised with X-gal in developing structures as described previously (Dingermann et al., 1989).

#### Knockout constructs for *Pvio acaA*, *acrA*, *carA* and *pdsA*

To disrupt *Pvio acaA*, an *acaA* fragment was amplified from *Pvio* gDNA using primers Pv-aca-51S2 and Pv-aca-31K that harbour *Sac*II and *Kpn*I sites (Table S1), respectively. After digestion, the fragment was cloned into *Sac*II/*Kpn*I digested pBluescript SK+, which was next digested with *Bam*HI and *Sal*I. The LoxP-NeoR cassette was excised from pLoxNeoIII (Kawabe et al., 2012) with *Bam*HI/*Sal*I and ligated into the digested acaA-pBluescript vector. This yielded pPv-acaA-KO in which LoxP-NeoR was flanked by 1870 bp of 5′UTR and 5′ *acaA* sequence and 1825 bp of 3′ *acaA* sequence (Fig. S2A).

To disrupt *Pvio acrA*, two *acrA* fragments, KO-A and KO-B, were amplified from *Pvio* genomic DNA using primer pair Pv-ACB-51K/Pv-ACB-31S with *Kpn*I and *Sac*II sites for KO-A and Pv-ACB-52 K/Pv-ACB-32 with a *Kpn*I site for KO-B (Table S1). After *Kpn*I/*Sac*II digestion, KO-A was ligated into *Kpn*I/*Sac*II digested pBluescript SK+, which was next digested with *Kpn*I/*Xba*I and ligated to LoxP-NeoR, which was excised from pLoxNeoIII with *Kpn*I/*Xba*I. Fragment KO-B was digested with *Kpn*I/*Xho*I and cloned into this vector, yielding vector pPv-acrA-KO with LoxP-NeoR flanked by 1483 bp 5′*acrA* sequence and 1373 bp 3′*acrA* and 3′UTR sequence (Fig. S2B).

The linearized KO vectors were transformed in *Pvio* (Narita et al., 2020). Genomic DNA was isolated from G418 resistant clones and screened by PCR to diagnose gene disruption by homologous recombination (Fig. S2). To generate a double *acaAˉacrAˉ* knock-out, *acaAˉ* cells were transformed with pA15NLS.Cre for transient expression of Cre-recombinase (Faix et al., 2004). G418 sensitive clones were selected and transformed with pPv-acrA-KO and diagnosed for *acrA* gene disruption by PCR (Fig. S2B).

To disrupt *Pvio carA* (Pvio_g6080, KAF2072602), two *carA* fragments, A and B, were amplified from *Pvio* gDNA using primer pair Pv-cAR-51K/Pv-cAR-31C with *Kpn*I and *Cla*I sites for A and Pv-cAR-52B/Pv-cAR-32X with *Bam*HI and *Xba*I sites for B (Table S1). After digestion with *Kpn*I and *Cla*I, fragment A was ligated into *Kpn*I/*Cla*I digested pLoxNeoIII. Fragment B was digested with *Bam*HI/*Xba*I and cloned into this vector, yielding vector pPv-carA-KO in which LoxP-NeoR is flanked by 1183 bp 5′UTR and 5′*carA* sequence and 1068 bp 3′ *carA* and 3′UTR sequence (Fig. S5A).

To disrupt *Pvio pdsA* (Pvio_g5708, KAF2072968), two *pdsA* fragments, A and B with internal *Hind*III and *Sac*I sites, respectively, were amplified from *Pvio* gDNA using primer pair Pv-pdsA-51K with *Kpn*I site and Pv-pdsA-31 for A and Pv-pdsA-52B with *Bam*HI site and Pv-pdsA-32 for B (Table S1). Fragment B was digested with *Bam*HI/*Sac*I and ligated into *Bam*HI/*Sac*I digested pLoxNeoIII. Fragment A was digested with *Kpn*I/*Hind*III and cloned into this vector, yielding vector pPv-pdsA-KO that contained 1391 bp 5′UTR and 5′*pdsA* sequence and 1205 bp 3′*pdsA* and 3′UTR sequence (Fig. S5B).

#### Complementation of adenylate cyclase knock-outs with *acaA* or *acrA*

To express *Pvio acaA* from its own promoter, a 5833 bp segment containing the *Pv-acaA* promoter, coding and terminator regions was amplified from gDNA in two fragments, A and B, using primer set Pv-aca-P51K (with *Kpn*I site) and Pv-aca-C31 for A and Pv-aca-C51 and Pv-aca-31C (with *Cla*I site) for B. The fragments were cloned into pCR-BluntII-TOPO for sequence validation. Using an *acaA* internal *Bam*HI site, fragment A was isolated from its TOPO plasmid using *Kpn*I and *Bam*HI and ligated into the *Kpn*I/*Bam*HI digested plasmid that contained fragment B, thus reconstructing the entire 5.8 kb *acaA* genomic segment. This segment was excised with *Kpn*I/*Cla*I and ligated into *Kpn*I/*Cla*I digested vector pHygTm(plus)/pG7 (http://dictybase.org/db/cgi-bin/dictyBase/SC/plasmid_details.pl?id=453), which contains a hygromycin resistance cassette, yielding vector pPv-acaA-Exp.

To express *Pvio acrA* from its own promoter, a segment containing the *acrA* coding region, part of its promoter and the terminator were amplified from gDNA using primers Pv-ACB-P51X/Pv-ACB-31S. The fragment was digested with *Spe*I/*Sac*II and cloned into pBluescript SK+ for sequence validation, yielding plasmid pBs-PvAcrA. The remaining part of the promoter region was excised with *Xba*I/*Sma*I from pPv-acrA-LacZ (see above) and ligated into the *Xba*I/*Sma*I digested vector pHygTm(plus)/pG7, which was subsequently digested with *Sma*I and *Spe*I. The PvAcrA fragment from pBs-PvAcrA was excised with *Spe*I/*Hpa*I and ligated into the *Spe*I/*Sma*I digested vector, yielding pPv-acrA-Exp, which now harboured a 6.4 kb region encompassing the *acrA* promoter, coding region and terminator. pPv-acrA-Exp was introduced into both *acrAˉ* and *acaAˉ/acrAˉ* cells by electroporation and pPv-acaA-Exp into *acaAˉ/acrAˉ* only. Transformants were incubated with autoclaved *Klebsiella aerogenes* in 10% HL5 with 30 μg/ml of hygromycin in petri dishes for 48 h, and next distributed with *E. coli* on 1/5th SM plates supplemented with 30 µg/ml of hygromycin. The plasmids and knock-out cell lines prepared for the study are in the *Dictyostelium* Stock Center http://dictybase.org/StockCenter/StockCenter.html.

### Data analysis

Quantitative data were collected in Excel (Microsoft). Statistical analysis and graph preparation was performed in Sigmaplot v14.5 (Systat Software, Inc.).

## Supplementary Material

10.1242/biolopen.059728_sup1 Supplementary information

## References

[BIO059728C1] Alvarez-Curto, E., Rozen, D. E., Ritchie, A. V., Fouquet, C., Baldauf, S. L. and Schaap, P. (2005). Evolutionary origin of cAMP-based chemoattraction in the social amoebae. *Proc. Natl. Acad. Sci. USA* 102, 6385-6390. 10.1073/pnas.0502238102 15851658 PMC1088387

[BIO059728C2] Alvarez-Curto, E., Saran, S., Meima, M., Zobel, J., Scott, C. and Schaap, P. (2007). cAMP production by adenylyl cyclase G induces prespore differentiation in Dictyostelium slugs. *Development* 134, 959-966. 10.1242/dev.02775 17267449 PMC2176081

[BIO059728C3] Araki, T. and Saito, T. (2019). Small molecules and cell differentiation in Dictyostelium discoideum. *Int. J. Dev. Biol.* 63, 429-438. 10.1387/ijdb.190192ts 31840781

[BIO059728C4] Asghar, A., Groth, M., Siol, O., Gaube, F., Enzensperger, C., Glockner, G. and Winckler, T. (2011). Developmental gene regulation by an ancient intercellular communication system in social amoebae. *Protist* 163, 25-37. 10.1016/j.protis.2010.12.002 21371934

[BIO059728C5] Brefeld, O. (1884). Polysphondylium violaceum und Dictyostelium mucoroides nebst Bemerkuengen zur Systematik der Schleimpilze. *Unters. Gesammtgeb. Mykol*. 6, 1-34.

[BIO059728C6] Brown, M. W., Kolisko, M., Silberman, J. D. and Roger, A. J. (2012). Aggregative multicellularity evolved independently in the Eukaryotic Supergroup Rhizaria. *Curr. Biol.* 22, 1123-1127. 10.1016/j.cub.2012.04.021 22608512

[BIO059728C7] Chen, Z. H. and Schaap, P. (2012). The prokaryote messenger c-di-GMP triggers stalk cell differentiation in *Dictyostelium*. *Nature* 488, 680-683. 10.1038/nature11313 22864416 PMC3939355

[BIO059728C8] Chen, Z. H., Singh, R., Cole, C., Lawal, H. M., Schilde, C., Febrer, M., Barton, G. J. and Schaap, P. (2017). Adenylate cyclase A acting on PKA mediates induction of stalk formation by cyclic diguanylate at the Dictyostelium organizer. *Proc. Natl. Acad. Sci. USA* 114, 516-521. 10.1073/pnas.1608393114 28057864 PMC5255622

[BIO059728C9] Clarke, M., Lohan, A. J., Liu, B., Lagkouvardos, I., Roy, S., Zafar, N., Bertelli, C., Schilde, C., Kianianmomeni, A., Burglin, T. R. et al. (2013). Genome of *Acanthamoeba castellanii* highlights extensive lateral gene transfer and early evolution of tyrosine kinase signaling. *Genome Biol.* 14, R11. 10.1186/gb-2013-14-2-r11 23375108 PMC4053784

[BIO059728C10] Dingermann, T., Reindl, N., Werner, H., Hildebrandt, M., Nellen, W., Harwood, A., Williams, J. and Nerke, K. (1989). Optimization and in situ detection of *Escherichia coli* beta-galactosidase gene expression in *Dictyostelium discoideum*. *Gene* 85, 353-362. 10.1016/0378-1119(89)90428-9 2516830

[BIO059728C11] Du, Q., Schilde, C., Birgersson, E., Chen, Z. H., Mcelroy, S. and Schaap, P. (2014). The cyclic AMP phosphodiesterase RegA critically regulates encystation in social and pathogenic amoebas. *Cell. Signal.* 26, 453-459. 10.1016/j.cellsig.2013.10.008 24184654 PMC3906536

[BIO059728C12] Du, Q., Kawabe, Y., Schilde, C., Chen, Z. H. and Schaap, P. (2015). The evolution of aggregative multicellularity and cell-cell communication in the Dictyostelia. *J. Mol. Biol*. 427, 3722-3733. 10.1016/j.jmb.2015.08.008 26284972 PMC5055082

[BIO059728C13] Faix, J., Kreppel, L., Shaulsky, G., Schleicher, M. and Kimmel, A. R. (2004). A rapid and efficient method to generate multiple gene disruptions in Dictyostelium discoideum using a single selectable marker and the Cre-loxP system. *Nucleic Acids Res.* 32, e143. 10.1093/nar/gnh136 15507682 PMC528815

[BIO059728C14] Funamoto, S., Anjard, C., Nellen, W. and Ochiai, H. (2003). cAMP-dependent protein kinase regulates *Polysphondylium pallidum* development. *Differentiation* 71, 51-61. 10.1046/j.1432-0436.2003.700605.x 12558603

[BIO059728C15] Gold, D. A., Katsuki, T., Li, Y., Yan, X., Regulski, M., Ibberson, D., Holstein, T., Steele, R. E., Jacobs, D. K. and Greenspan, R. J. (2019). The genome of the jellyfish Aurelia and the evolution of animal complexity. *Nat. Ecol. Evol.* 3, 96-104. 10.1038/s41559-018-0719-8 30510179

[BIO059728C16] Harwood, A. J. and Drury, L. (1990). New vectors for expression of the *E.coli lacZ* gene in *Dictyostelium*. *Nucl. Acids Res*. 18, 4292. 10.1093/nar/18.14.4292 2198542 PMC331230

[BIO059728C17] Harwood, A. J., Hopper, N. A., Simon, M.-N., Driscoll, D. M., Veron, M. and Williams, J. G. (1992). Culmination in *Dictyostelium* is regulated by the cAMP-dependent protein kinase. *Cell* 69, 615-624. 10.1016/0092-8674(92)90225-2 1586944

[BIO059728C18] Hillmann, F., Forbes, G., Novohradska, S., Ferling, I., Riege, K., Groth, M., Westermann, M., Marz, M., Spaller, T., Winckler, T. et al. (2018). Multiple roots of fruiting body formation in Amoebozoa. *Genome Biol. Evol.* 10, 591-606. 10.1093/gbe/evy011 29378020 PMC5804921

[BIO059728C19] Hirose, S., Katoh-Kurasawa, M. and Shaulsky, G. (2021). Cyclic AMP is dispensable for allorecognition in Dictyostelium cells overexpressing PKA-C. *J. Cell Sci.* 134, jcs258777. 10.1242/jcs.258777 34169317 PMC8325953

[BIO059728C20] Hopper, N. A., Harwood, A. J., Bouzid, S., Véron, M. and Williams, J. G. (1993). Activation of the prespore and spore cell pathway of *Dictyostelium* differentiation by cAMP-dependent protein kinase and evidence for its upstream regulation by ammonia. *EMBO J.* 12, 2459-2466. 10.1002/j.1460-2075.1993.tb05900.x 8508771 PMC413481

[BIO059728C21] Kalla, S. E., Queller, D. C., Lasagni, A. and Strassmann, J. E. (2011). Kin discrimination and possible cryptic species in the social amoeba Polysphondylium violaceum. *BMC Evol. Biol.* 11, 31. 10.1186/1471-2148-11-31 21272359 PMC3041686

[BIO059728C22] Kawabe, Y. and Schaap, P. (2022). Adenylate cyclase A amplification and functional diversification during *Polyspondylium pallidum* development. *EvoDevo* 13, 18. 10.1186/s13227-022-00203-7 36261860 PMC9583560

[BIO059728C23] Kawabe, Y., Kuwayama, H., Morio, T., Urushihara, H. and Tanaka, Y. (2002). A putative serpentine receptor gene *tasA* required for normal morphogenesis of primary stalk and branch structure in *Polysphondylium pallidum*. *Gene* 285, 291-299. 10.1016/S0378-1119(01)00887-3 12039057

[BIO059728C24] Kawabe, Y., Morio, T., James, J. L., Prescott, A. R., Tanaka, Y. and Schaap, P. (2009). Activated cAMP receptors switch encystation into sporulation. *Proc. Natl. Acad. Sci. USA* 106, 7089-7094. 10.1073/pnas.0901617106 19369200 PMC2678454

[BIO059728C25] Kawabe, Y., Weening, K. E., Marquay-Markiewicz, J. and Schaap, P. (2012). Evolution of self-organisation in Dictyostelia by adaptation of a non-selective phosphodiesterase and a matrix component for regulated cAMP degradation. *Development* 139, 1336-1345. 10.1242/dev.077099 22357931 PMC3294436

[BIO059728C26] Kawabe, Y., Schilde, C., Du, Q. and Schaap, P. (2015). A conserved signalling pathway for amoebozoan encystation that was co-opted for multicellular development. *Sci. Rep.* 5, 9644. 10.1038/srep09644 25881075 PMC4399386

[BIO059728C27] Kikuchi, H., Kubohara, Y., Nguyen, V. H., Katou, Y. and Oshima, Y. (2013). Novel chlorinated dibenzofurans isolated from the cellular slime mold, Polysphondylium filamentosum, and their biological activities. *Bioorg. Med. Chem.* 21, 4628-4633. 10.1016/j.bmc.2013.05.022 23746784

[BIO059728C28] Kin, K. and Schaap, P. (2021). Evolution of multicellular complexity in the Dictyostelid Social Amoebas. *Genes* 12, 487. 10.3390/genes12040487 33801615 PMC8067170

[BIO059728C29] King, N., Westbrook, M. J., Young, S. L., Kuo, A., Abedin, M., Chapman, J., Fairclough, S., Hellsten, U., Isogai, Y., Letunic, I. et al. (2008). The genome of the choanoflagellate Monosiga brevicollis and the origin of metazoans. *Nature* 451, 783-788. 10.1038/nature06617 18273011 PMC2562698

[BIO059728C30] Kondo, A. P., Narita, T. B., Murata, C., Ogura, T., Mikagi, A., Usuki, T. and Saito, T. (2019). 4-Methyl-5-Pentylbenzene-1,3-Diol regulates chemotactic cell aggregation and spore maturation via different mechanisms in Dictyostelium discoideum. *Curr. Microbiol.* 76, 376-381. 10.1007/s00284-019-01639-2 30710153

[BIO059728C31] König, S. G. and Nedelcu, A. M. (2020). The genetic basis for the evolution of soma: mechanistic evidence for the co-option of a stress-induced gene into a developmental master regulator. *Proc. Biol. Sci.* 287, 20201414. 10.1098/rspb.2020.1414 33259762 PMC7739920

[BIO059728C32] Loomis, W. F. (2014). Cell signaling during development of *Dictyostelium*. *Dev. Biol*. 391, 1-16. 10.1016/j.ydbio.2014.04.001 24726820 PMC4075484

[BIO059728C33] Lyons, N. A. and Kolter, R. (2015). On the evolution of bacterial multicellularity. *Curr. Opin. Microbiol.* 24, 21-28. 10.1016/j.mib.2014.12.007 25597443 PMC4380822

[BIO059728C34] Narita, T. B., Kawabe, Y., Kin, K., Gibbs, R. A., Kuspa, A., Muzny, D. M., Richards, S., Strassmann, J. E., Sucgang, R., Worley, K. C. et al. (2020). Loss of the Polyketide Synthase StlB Results in Stalk Cell Overproduction in Polysphondylium violaceum. *Genome Biol. Evol.* 12, 674-683. 10.1093/gbe/evaa079 32386295 PMC7259674

[BIO059728C35] Patel, H., Guo, K., Parent, C., Gross, J., Devreotes, P. N. and Weijer, C. J. (2000). A temperature-sensitive adenylyl cyclase mutant of *Dictyostelium*. *EMBO J.* 19, 2247-2256. 10.1093/emboj/19.10.2247 10811616 PMC384365

[BIO059728C36] Pitt, G. S., Milona, N., Borleis, J., Lin, K. C., Reed, R. R. and Devreotes, P. N. (1992). Structurally distinct and stage-specific adenylyl cyclase genes play different roles in *Dictyostelium* development. *Cell* 69, 305-315. 10.1016/0092-8674(92)90411-5 1348970

[BIO059728C37] Ritchie, A. V., Van Es, S., Fouquet, C. and Schaap, P. (2008). From drought sensing to developmental control: evolution of cyclic AMP signaling in social amoebas. *Mol. Biol. Evol.* 25, 2109-2118. 10.1093/molbev/msn156 18640994 PMC2535757

[BIO059728C38] Romeralo, M., Skiba, A., Gonzalez-Voyer, A., Schilde, C., Lawal, H., Kedziora, S., Cavender, J. C., Glockner, G., Urushihara, H. and Schaap, P. (2013). Analysis of phenotypic evolution in Dictyostelia highlights developmental plasticity as a likely consequence of colonial multicellularity. *Proc. Biol. Sci.* 280, 20130976. 10.1098/rspb.2013.0976 23782883 PMC3712420

[BIO059728C39] Rossier, C., Gerisch, G., Malchow, D. and Eckstein, F. (1978). Action of a slowly hydrolysable cyclic AMP analogue on developing cells of *Dictyostelium discoideum*. *J.Cell Sci*. 35, 321-338. 10.1242/jcs.35.1.321 217886

[BIO059728C40] Saito, T., Iijima, T., Koyama, K., Shinagawa, T., Yamanaka, A., Araki, T., Suzuki, N., Usuki, T. and Kay, R. R. (2022). Generating polyketide diversity in Dictyostelium: a Steely hybrid polyketide synthase produces alternate products at different developmental stages. *Proc. Biol. Sci.* 289, 20221176. 10.1098/rspb.2022.1176 36126683 PMC9489281

[BIO059728C41] Sasaki, H., Kubohara, Y., Ishigaki, H., Takahashi, K., Eguchi, H., Sugawara, A., Oshima, Y. and Kikuchi, H. (2020). Two new terpenes isolated from dictyostelium cellular slime molds. *Molecules* 25, 2895. 10.3390/molecules25122895 32585998 PMC7356884

[BIO059728C42] Schaap, P. (1985). cAMP relay during early culmination of Dictyostelium minutum. *Differentiation* 28, 205-208. 10.1111/j.1432-0436.1985.tb00826.x 2985287

[BIO059728C43] Schaap, P. and Van Driel, R. (1985). Induction of post-aggregative differentiation in *Dictyostelium discoideum* by cAMP. Evidence of involvement of the cell surface cAMP receptor. *Exp. Cell Res*. 159, 388-396. 10.1016/S0014-4827(85)80012-4 2993006

[BIO059728C44] Schaap, P., Konijn, T. M. and Van Haastert, P. J. M. (1984). cAMP pulses coordinate morphogenetic movement during fruiting body formation of *Dictyostelium minutum*. *Proc. Natl. Acad. Sci. USA* 81, 2122-2126. 10.1073/pnas.81.7.2122 16593448 PMC345449

[BIO059728C45] Schaap, P., Winckler, T., Nelson, M., Alvarez-Curto, E., Elgie, B., Hagiwara, H., Cavender, J., Milano-Curto, A., Rozen, D. E., Dingermann, T. et al. (2006). Molecular phylogeny and evolution of morphology in the social amoebas. *Science* 314, 661-663. 10.1126/science.1130670 17068267 PMC2173941

[BIO059728C46] Schaap, P., Barrantes, I., Minx, P., Sasaki, N., Anderson, R. W., Benard, M., Biggar, K. K., Buchler, N. E., Bundschuh, R., Chen, X. et al. (2015). The Physarum polycephalum genome reveals extensive use of prokaryotic two-component and Metazoan-type tyrosine kinase signaling. *Genome Biol. Evol.* 8, 109-125. 10.1093/gbe/evv237 26615215 PMC4758236

[BIO059728C47] Schilde, C., Skiba, A. and Schaap, P. (2014). Evolutionary reconstruction of pattern formation in 98 Dictyostelium species reveals that cell-type specialization by lateral inhibition is a derived trait. *EvoDevo* 5, 34. 10.1186/2041-9139-5-34 25904998 PMC4406040

[BIO059728C48] Schilde, C., Lawal, H. M., Kin, K., Shibano-Hayakawa, I., Inouye, K. and Schaap, P. (2019). A well supported multi gene phylogeny of 52 dictyostelia. *Mol. Phylogenet. Evol.* 134, 66-73. 10.1016/j.ympev.2019.01.017 30711536 PMC6430600

[BIO059728C49] Shimomura, O., Suthers, H. L. B. and Bonner, J. T. (1982). Chemical identity of the acrasin of the cellular slime mold *Polysphondylium violaceum*. *Proc. Natl. Acad. Sci. USA* 79, 7376-7379. 10.1073/pnas.79.23.7376 6961416 PMC347342

[BIO059728C50] Singer, G., Araki, T. and Weijer, C. J. (2019). Oscillatory cAMP cell-cell signalling persists during multicellular Dictyostelium development. *Commun. Biol.* 2, 139. 10.1038/s42003-019-0371-0 31044164 PMC6478855

[BIO059728C51] Soderbom, F., Anjard, C., Iranfar, N., Fuller, D. and Loomis, W. F. (1999). An adenylyl cyclase that functions during late development of *Dictyostelium*. *Development* 126, 5463-5471. 10.1242/dev.126.23.5463 10556070

[BIO059728C52] Srivastava, M., Simakov, O., Chapman, J., Fahey, B., Gauthier, M. E., Mitros, T., Richards, G. S., Conaco, C., Dacre, M., Hellsten, U. et al. (2010). The Amphimedon queenslandica genome and the evolution of animal complexity. *Nature* 466, 720-726. 10.1038/nature09201 20686567 PMC3130542

[BIO059728C53] Sucgang, R., Weijer, C. J., Siegert, F., Franke, J. and Kessin, R. H. (1997). Null mutations of the *Dictyostelium* cyclic nucleotide phosphodiesterase gene block chemotactic cell movement in developing aggregates. *Dev. Biol*. 192, 181-192. 10.1006/dbio.1997.8720 9405107

[BIO059728C54] Suga, H., Chen, Z., De Mendoza, A., Sebe-Pedros, A., Brown, M. W., Kramer, E., Carr, M., Kerner, P., Vervoort, M., Sanchez-Pons, N. et al. (2013). The Capsaspora genome reveals a complex unicellular prehistory of animals. *Nat. Commun.* 4, 2325. 10.1038/ncomms3325 23942320 PMC3753549

[BIO059728C55] Sun, T. J., Van Haastert, P. J. M. and Devreotes, P. N. (1990). Surface cAMP receptors mediate multiple responses during development in Dictyostelium: evidenced by antisense mutagenesis. *J.Cell Biol*. 110, 1549-1554. 10.1083/jcb.110.5.1549 1692327 PMC2200165

[BIO059728C56] Tice, A. K., Silberman, J. D., Walthall, A. C., Le, K. N., Spiegel, F. W. and Brown, M. W. (2016). Sorodiplophrys stercorea: another Novel Lineage of Sorocarpic Multicellularity. *J. Eukaryot. Microbiol.* 63, 623-628. 10.1111/jeu.12311 26940948

[BIO059728C57] Tsujioka, M., Yamamoto, T., Thompson, C. R., Kay, R. R. and Maeda, M. (2004). Novel development rescuing factors (DRFs) secreted by the developing Dictyostelium cells, that are involved in the restoration of a mutant lacking MAP-kinase ERK2. *Zoolog. Sci.* 21, 829-834. 10.2108/zsj.21.829 15333995

[BIO059728C58] Van Es, S., Virdy, K. J., Pitt, G. S., Meima, M., Sands, T. W., Devreotes, P. N., Cotter, D. A. and Schaap, P. (1996). Adenylyl cyclase G, an osmosensor controlling germination of *Dictyostelium* spores. *J. Biol. Chem*. 271, 23623-23625. 10.1074/jbc.271.39.23623 8798577

[BIO059728C59] Van Haastert, P. J. M. and Kien, E. (1983). Binding of cAMP derivatives to Dictyostelium discoideum cells. Activation mechanism of the cell surface cAMP receptor. *J.Biol.Chem*. 258, 9636-9642. 10.1016/S0021-9258(17)44544-3 6309778

[BIO059728C60] Wang, M., Van Driel, R. and Schaap, P. (1988). Cyclic AMP-phosphodiesterase induces dedifferentiation of prespore cells in Dictyostelium discoideum slugs: evidence that cyclic AMP is the morphogenetic signal for prespore differentiation. *Development* 103, 611-618. 10.1242/dev.103.3.611

