# Supplementary material for: Development of the dictyostelid Polysphondylium violaceum does not require secreted cAMP
Source: Biol Open. 2023 Feb 2;12(2):bio059728. doi: 10.1242/bio.059728 (PMC9922732; doi:10.1242/bio.059728)
Supplement: Supplementary information [file biolopen-12-059728-s1.pdf]

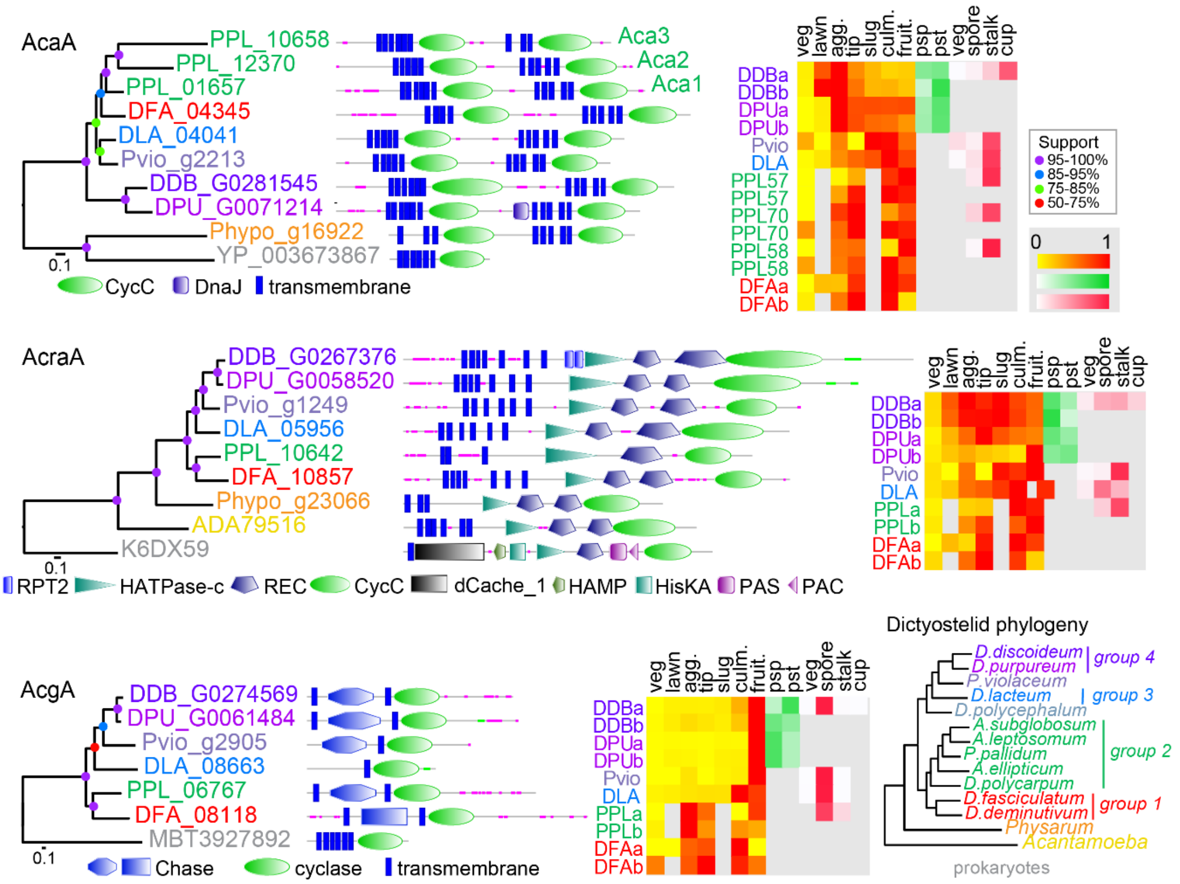

Fig. S1. Adenylate cyclase genes in group-representative genomes

All homologs of *Ddis* AcaA, AcrA and AcgA were identified by BlastP search of taxon-group specific dictyostelid proteomes and the proteomes of the solitary Amoebozoa *Physarum polycephalum* (Phypo) and *Acanthamoeba castellani*. The most closely related homologs outside Amoebozoa were identified by BlastP search of all non-redundant sequences in Genbank and proved all to be prokaryote adenylate cyclases. Phylogenetic trees were inferred with MrBayes (Ronquist and Huelsenbeck, 2003) from the aligned protein sequences for each cyclase. The gene identifiers (NCBI https://www.ncbi.nlm.nih.gov/ for prokaryote and *Acanthamoeba* genes, Dictybase http://dictybase.org/ for *Ddis* and *Dpur*, SACGB http://sacgb.leibniz-fli.de/cgi/index.pl for *P. pallidum*, *D. lacteum* and *D. fasciculatum* and original source locus tags for *Pvio* and *Physarum polycephalum* genes) are colour coded to reflect the taxonomic affiliation of the host species (inset at lower right) Statistical support for the nodes is indicated by coloured dots. The tree was annotated with the functional domain architecture of the proteins as analysed in SMART (Schultz et al., 1998) and with heatmaps of relative transcript levels at specific developmental stages or in specific cell types, which were retrieved from published RNA sequencing experiments (Forbes et al., 2019; Gloeckner et al., 2016; Kin et al., 2018; Parikh et al., 2010). The Dictyostelid phylogeny inferred from 47 concatenated proteins is retrieved from (Singh et al., 2016).

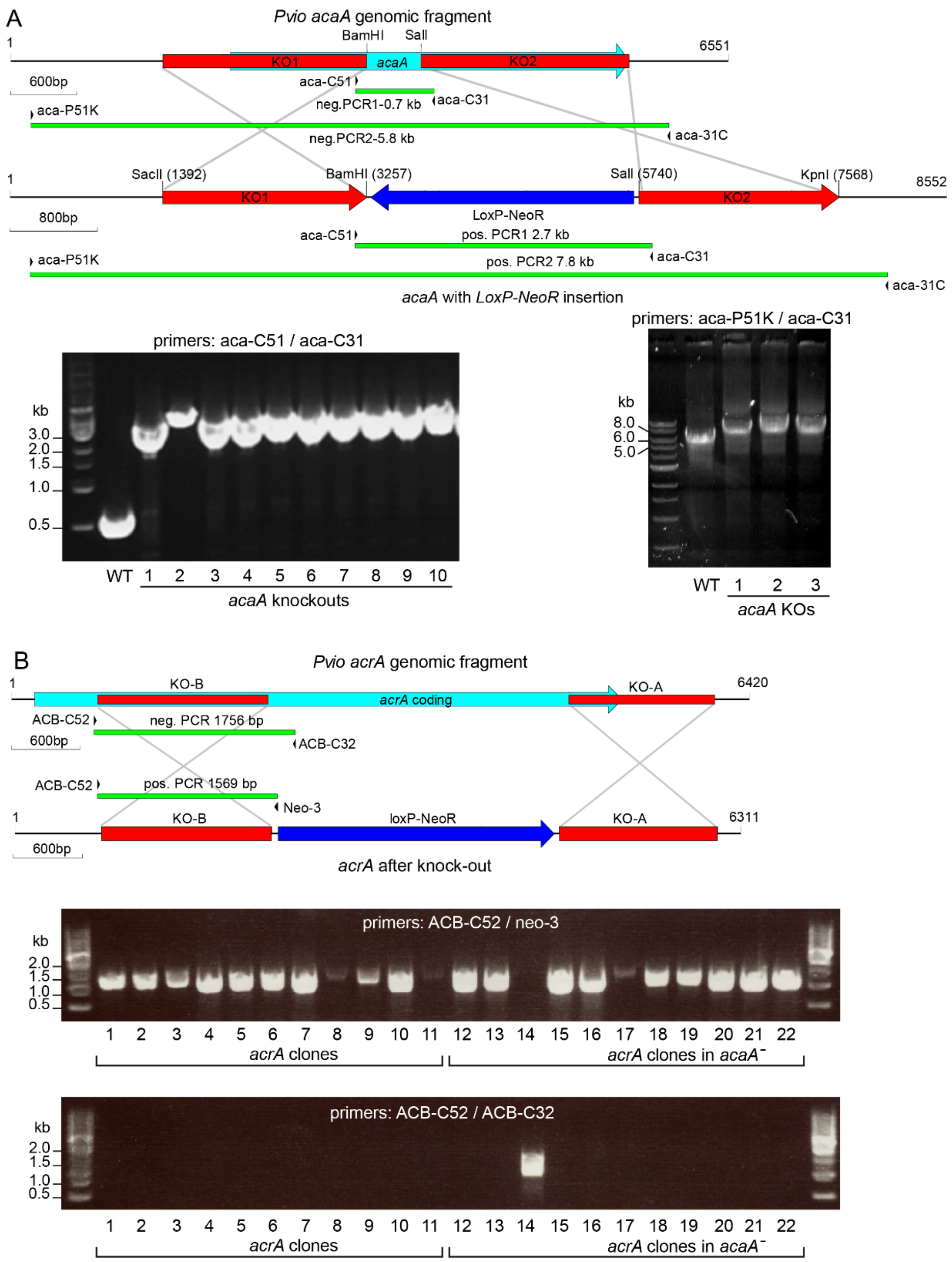

Fig. S2. Schematics and diagnoses of *Pvio acaA*, *acrA* and *acaAacrA* knock-outs *A. Pvio acaA knock-out.* The schematic shows a genomic fragment that contains the *acaA* gene before and after homologous recombination with the linearized pPpv-acaA-KO construct with the positions of primers used for diagnosis. Primer pair aca-C51/aca-C31 amplifies 0.7 or 2.7 kb products in wild-type (WT) or knock-outs respectively, while primer pair aca-P51K/aca- 31C amplify 5.8 kb or 7.8 kb products in WT or knock-outs, respectively. The gel images of the diagnostic PCRs show that all tested transformed clones were knock-outs.

B. Pvio acrA and acaAacrA knockouts. Both wild-type and *acaA*$^-$ cells were transformed with pPv-acrA-KO. Schematic of the *acrA* genomic region before and after recombination with pPv-acrA-KO. Primer pair ACB-C52/ACB-C32 amplifies a 1.8 kb fragment in WT only, while primer pair ACB-C52/ Neo-3 amplifies a 1.6 kb fragment only in knock-outs. The gel images show that except for *acaA*$^-$ *acrA* clone 14, all transformed clones were knock-outs.

Biology Open • Supplementary information

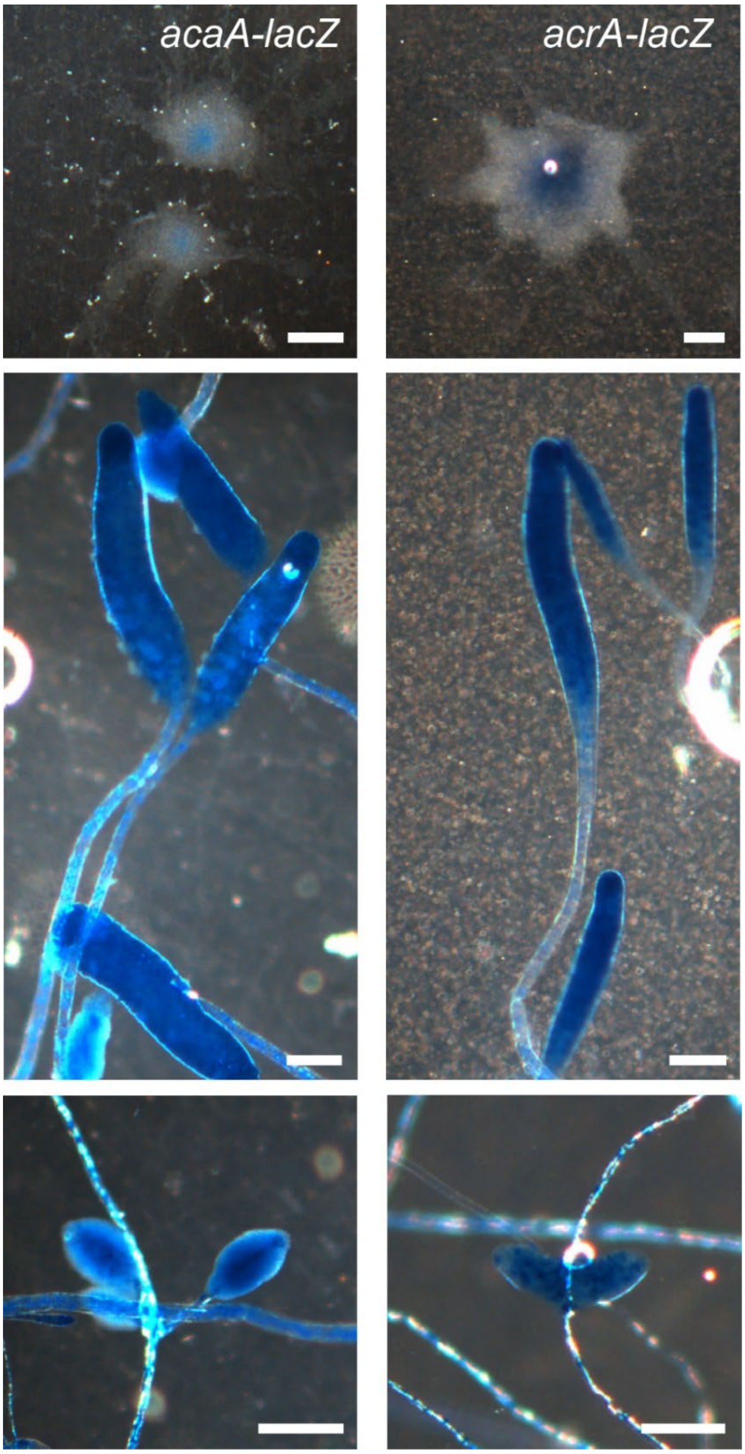

Fig. S3. Expression patterns of *acaA and acrA* genes.

Intergenic regions upstream of the start-codons of the *Pvio acaA* and *acrA* genes were amplified, fused to *lacZ* and transformed into *Pvio* wild-type cells. The cells were developed on KK2 agar and developing structures were fixed and stained with X-gal. Top panels: aggregates; middle panels: primary sorogens; bottom panels: secondary sorogens. Bars: 0.1 mm.

Biology Open • Supplementary information

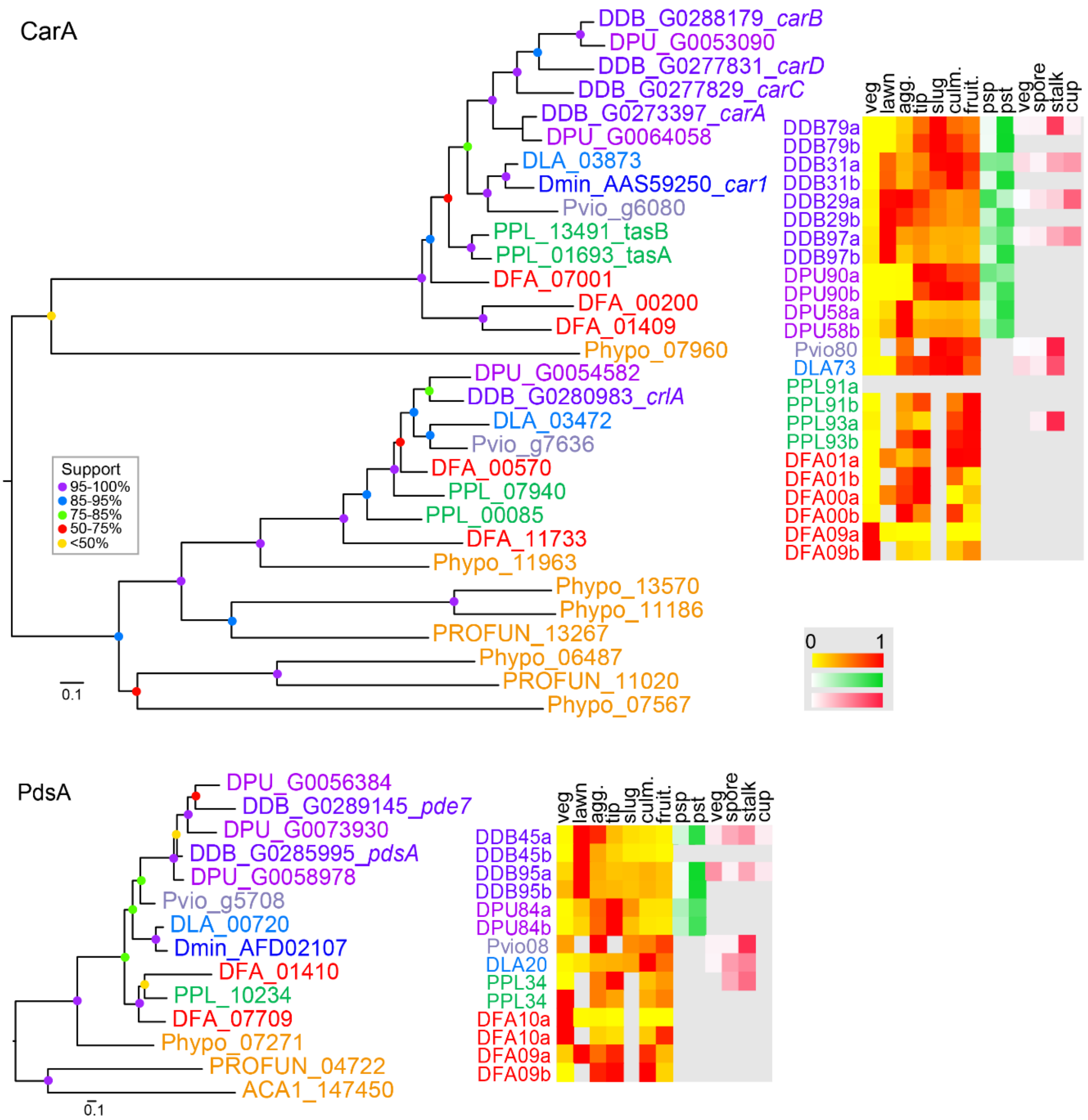

Fig. S4. Conservation of Cars and PdsA across Dictyostelia

Homologs of *Ddis* CarA and PdsA were retrieved by BlastP search from taxon group representative dictyostelid proteomes and proteomes of the solitary Amoebozoa *Physarum polycephalum* (Phypo), *Protostelium fungivorum* (PROFUN). Phylogenetic trees were inferred from the aligned protein sequences, using IQ-TREE (Trifinopoulos et al., 2016). For the CarA tree only the most closely related clade of car-like (crl) receptors is included in the tree. The trees are annotated with the developmental and cell-type specific expression profiles of the dictyostelid *carA* and *pdsA* homologs as in Figure S1.

Biology Open • Supplementary information

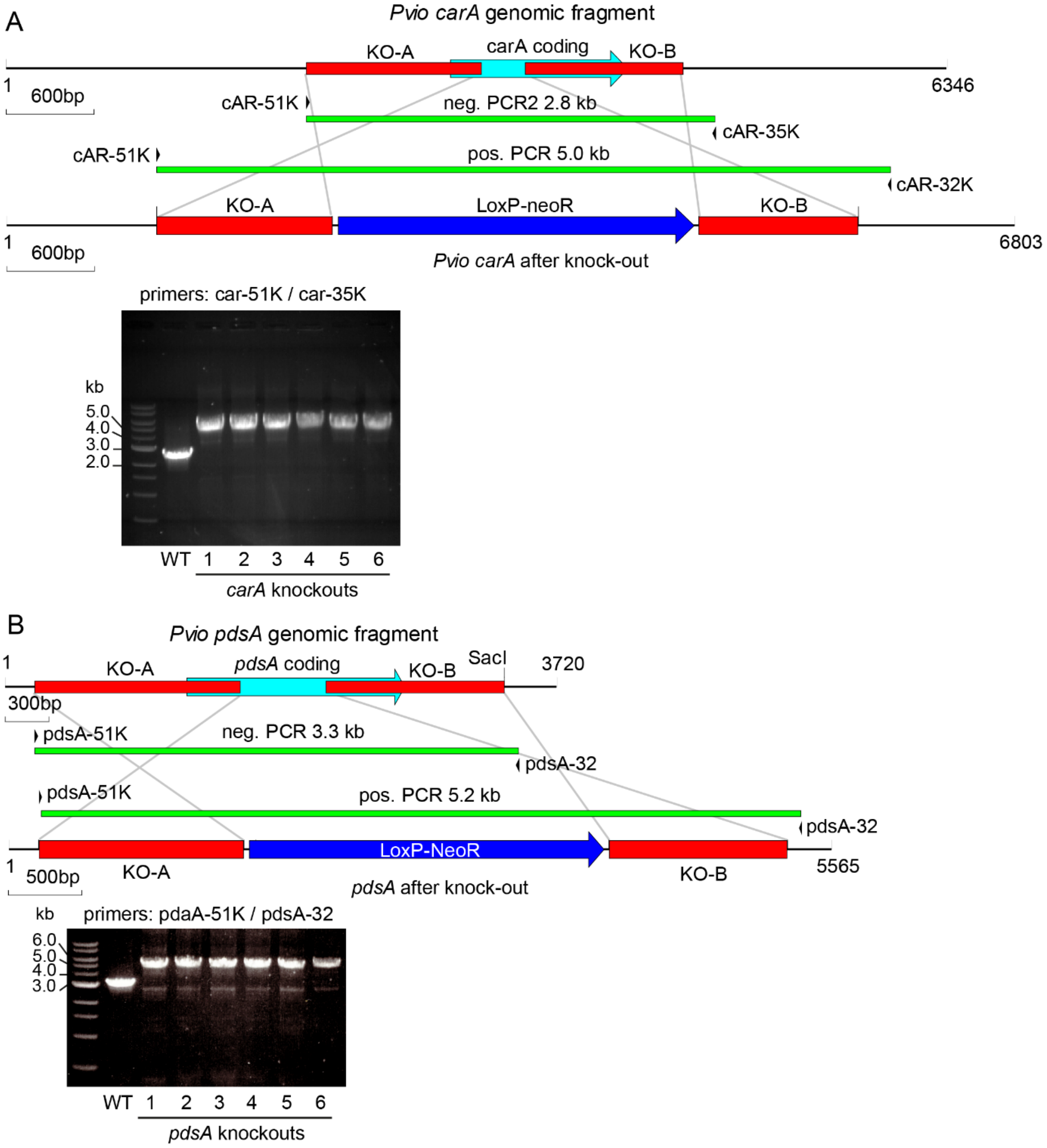

Fig. S5. Schematics and diagnoses of *P.vio carA and pdsA* knock-outs

A. Pvio carA knock-out. Schematic of a *carA* (Pvio_g6080, KAF2072602.1) genomic fragment before and after insertion of the LoxP-neoR cassette with the position of the diagnostic primers and expected PCR products before (neg.) and after (pos.) insertion. The gel image of the diagnostic PCR in wild-type and transformed clones show all clones were knock-outs.

B. Pvio pdsA knock-out. Schematic of a *pdsA* (Pvio_g5708, KAF2072968.1) genomic fragment before and after knockout. Primers pdsA-51K and pdsA-32 amplify a 3.3 kb product in wild-type and off-target integrants and a 5.2 kb product in *pdsA* knock-outs. The gel image shows that all tested transformed clones were knock-outs.

Biology Open • Supplementary information

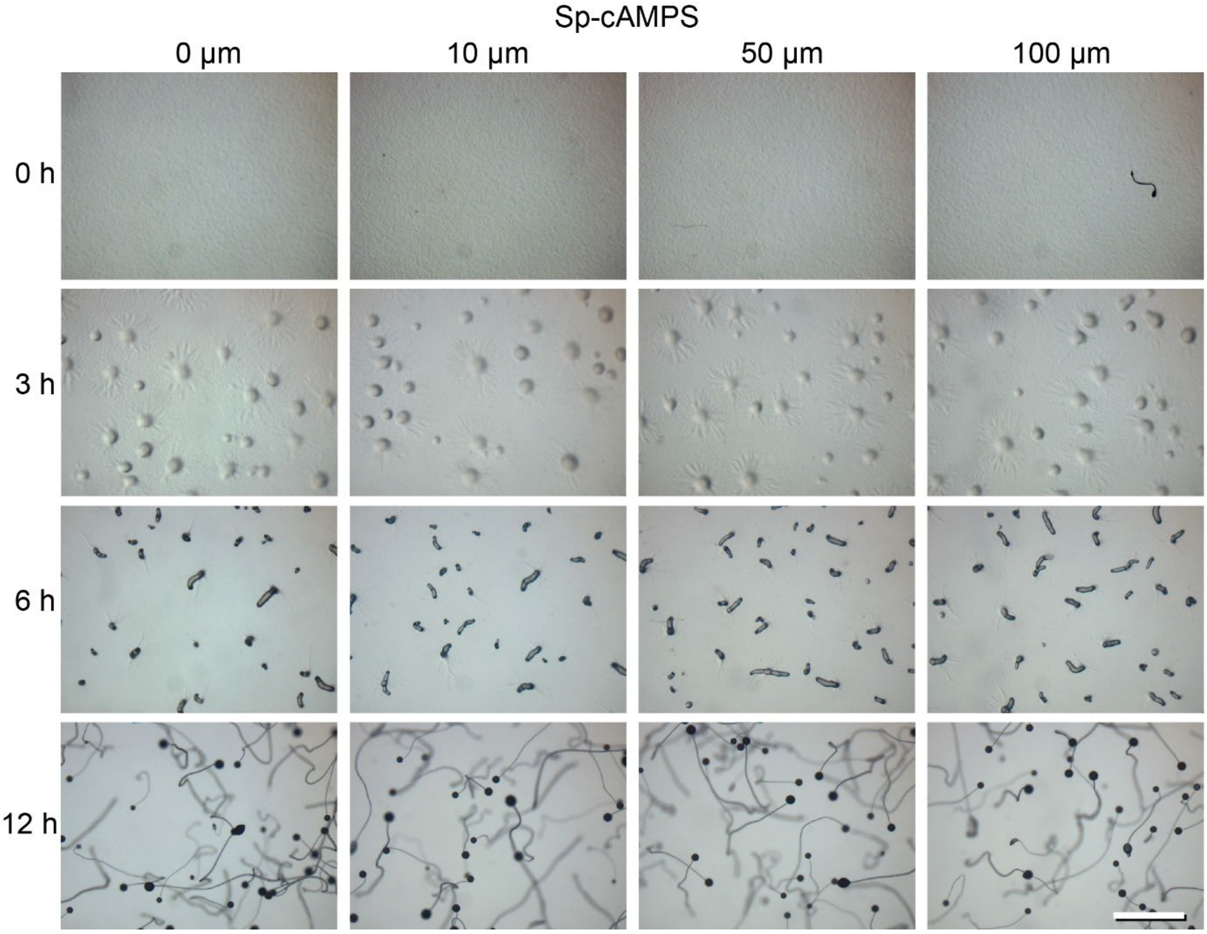

Fig. S6. Effect of Sp-cAMPS on *Pvio* development

Pvio cells were distributed at 10^6 cells/cm^2 on KK2 agar containing the indicated concentrations of Sp-cAMPS. At the indicated time points cells were photographed under transmitted light. Bar: 1 mm. A repeat experiment using only 0 and 100 µM Sp-cAMPS yielded the same result.

Biology Open • Supplementary information

```
TRINITY_DN17441   --------------------------------------------------------------------------------
TRINITY_DN9159    ------------------------------------------TTTTTTGGTGGTTGTTTTTTTTATCAACTATACCCAAA
TRINITY_DN6833    --------TATTATTTTTTTCAACATTTGAAAATAATAATAATTTTTTGGTGTTGGTTTTTTTTATCAACTATACCCAAA
Pvio_g6080        TAGCGTATTATTATTTTTTTCAACATTTGAAAATAATAATAATTTTTTGGTGTTGGTTTTTTTTATCAACTATACCCAAA

TRINITY_DN17441   --------------------------------------------------------------------------------
TRINITY_DN9159    AGTATATCAAACCTAGTTAACAGACAAACAGACTCACAGATCAATCACATAAAATGGGTCAACCCATTTCAATTTCGAAT
TRINITY_DN6833    AGTATATCAAACCTAGTTAACAGACAAACAGACTCACAGATCAATCACATAAAATGGGTCAACCCATTTCAATTTCGAAT
Pvio_g6080        AGTATATCAAACCTAGTTAACAGACAAACAGACTCACAGATCAATCACATAAAATGGGTCAACCCATTTCAATTTCGAAT

TRINITY_DN17441   --------------------------------------------------------------------------------
TRINITY_DN9159    GATGAAATTATCGTATCCTATACACTTTTGCTATTTGCCGATTTTACATCTATTATTGGATGTGGTATTGTATTGATAGC
TRINITY_DN6833    GATGAAATTATCGTATCCTATACACTTTTGCTATTTGCCGATTTTACATCTATTATTGGATGTGGTATTGTATTGATAGC
Pvio_g6080        GATGAAATTATCGTATCCTATACACTTTTGCTATTTGCCGATTTTACATCTATTATTGGATGTGGTATTGTATTGATAGC

TRINITY_DN17441   --------------------------------------------------------------------------------
TRINITY_DN9159    TTTTTGGAGGTTACGATTACTAAGAAATCATGTGACAAAAGTTATAGCTTGTTTTTGCGGAACATCGGTAGCTAAGGATA
TRINITY_DN6833    TTTTTGGAGGTTACGATTACTAAGAAATCATGTGACAAAAGTTATAGCTTGTTTTTGCGGAACATCGGTAGCTAAGGATA
Pvio_g6080        TTTTTGGAGGTTACGATTACTAAGAAATCATGTGACAAAAGTTATAGCTTGTTTTTGCGGAACATCGGTAGCTAAGGATA

TRINITY_DN17441   --------------------------------------------------------------------------------
TRINITY_DN9159    TTCCCTCTACCATTTTAACCCTAACAAGATCCTCCCAGGGTATGGGTTTTCCCTGTTATTTTTATTCAATCTTAATTACC
TRINITY_DN6833    TTCCCTCTACCATTTTAACCCTAACAAGATCCTCCCAGGGTATGGGTTTTCCCTGTTATTTTTATTCAATCTTAATTACC
Pvio_g6080        TTCCCTCTACCATTTTAACCCTAACAAGATCCTCCCAGGGTATGGGTTTTCCCTGTTATTTTTATTCAATCTTAATTACC

TRINITY_DN17441   --------------------------------------------------------------------------------
TRINITY_DN9159    TATGGAAGTTTATCTTGTTGGATGTGGACACTCTGTTTAGGTAATTTTAATTTTATAATTGATTAGTTGACTCAAATATT
TRINITY_DN6833    TATGGAAGTTTATCTTGTTGGATGTGGACACTCTGTTTAGCTATT-----------------------------------
Pvio_g6080        TATGGAAGTTTATCTTGTTGGATGTGGACACTCTGTTTAGGTAATTTTAATTTTATAATTGATTAGTTGACTCAAATATT
```

Biology Open • Supplementary information

```
TRINITY_DN17441   --------------------------------------------------------------------------------
TRINITY_DN9159    GACATTATCACACTTTATCACAATAGCTATTAGTATTTATAGATTAATTGTTAAAAGAGATCCAGATGTAGAAAAGTATG
TRINITY_DN6833    -------------------------------AGTATTTATAGATTAATTGTTAAAAGAGATCCAGATGTAGAAAAGTATG
Pvio_g6080        GACATTATCACACTTTATCACAATAGCTATTAGTATTTATAGATTAATTGTTAAAAGAGATCCAGATGTAGAAAAGTATG

TRINITY_DN17441   --------------------------------------------------------------------------------
TRINITY_DN9159    AAAAATGGTACTATCTATCGTGTTGGGGAGCACCGGTGATTCCCACTATCATTATGTTGGCCAAAAATACAGTAATTTAT
TRINITY_DN6833    AAAAATGGTACTATCTATCGTGTTGGGGAGCACCGGTGATTCCCACTATCATTATGTTGGCCAAAAATACAGTAATTTAT
Pvio_g6080        AAAAATGGTACTATCTATCGTGTTGGGGAGCACCGGTGATTCCCACTATCATTATGTTGGCCAAAAATACAGTAATTTAT

TRINITY_DN17441   ----------------------------------TGGCC-------AAAAATACAGTAATTTATCCTTCTTTATTATATT
TRINITY_DN9159    CTTGGTAATTGGTGCTGGATTAGTGGAAAGCATATTGCATACAGATTTGTATTGTTTTACGTACCCTTCTTTTTTATTTT
TRINITY_DN6833    CTTGGTAATTGGTGCTGGATTAGTGGAAAGCATATTGCATACAGATTTGTATTGTTTTACGTACCCTTCTTTATTATATT
Pvio_g6080        CTTGGTAATTGGTGCTGGATTAGTGGAAAGCATATTGCATACAGATTTGTATTGTTTTACGTACCCTTCTTTATTATATT

TRINITY_DN17441   TTTCGTTTCTGCCATCTTGGTTGGCATTACCATGAGATACACCTACAATGTCATTCATAATGGCGTCAGTGACAATCGTG
TRINITY_DN9159    TTTCGTTTCTCCCATCTTGGTTG---------------------------------------------------------
TRINITY_DN6833    TTTCGTTTCTGCCATCTTGGTTGGCATTACCATGAGATACACCTACAATGTCATTCATAATGGCGTCAGTGACAATCGTG
Pvio_g6080        TTTCGTTTCTGCCATCTTGGTTGGCATTACCATGAGATACACCTACAATGTCATTCATAATGGCGTCAGTGACAATCGTG

TRINITY_DN17441   ATAAACATTTTACATATCAATTTAAATTGGTCAACTATATTGTAGTCTTTCTTGTTTGTTGGTTGTTTGCCGTCATAAAC
TRINITY_DN9159    --------------------------------------------------------------------------------
TRINITY_DN6833    ATAAACATTTTACATATCAATTTAAATTGGTCAACTATATTGTAGTCTTTCTTGTTTGTTGGTTGTTTGCCGTCATAAAC
Pvio_g6080        ATAAACATTTTACATATCAATTTAAATTGGTCAACTATATTGTAGTCTTTCTTGTTTGTTGGTTGTTTGCCGTCATAAAC


TRINITY_DN17441   AGAATTTTAAATTCCATTGGTTACTATCCTACCGTACCCAATATTCTCCACACCTATCTCAGTGTATCACATGGCTTTTT
TRINITY_DN9159    --------------------------------------------------------------------------------
TRINITY_DN6833    AGAATTTTAAATTCCATTGGTTACTATCCTACCGTACCCAATATTCTCCACACCTATCTCAGTGTATCACATGGCTTTTT
Pvio_g6080        AGAATTTTAAATTCCATTGGTTACTATCCTACCGTACCCAATATTCTCCACACCTATCTCAGTGTATCACATGGCTTTTT

TRINITY_DN17441   TGCCAGCTGTGCTTTCATTTACAACAATCCATTGTTGTGGCGTTACTTGGGTTGCAAGGTATTAAAAGTGTTCGCAGCAT
TRINITY_DN9159    --------------------------------------------------------------------------------
TRINITY_DN6833    TGCCAGCTGTGCTTTCATTTACAACAATCCATTGTTGTGGCGTTACTTGGGTTGCAAGGTATTAAAAGTGTTCGCAGCAT
Pvio_g6080        TGCCAGCTGTGCTTTCATTTACAACAATCCATTGTTGTGGCGTTACTTGGGTTGCAAGGTATTAAAAGTGTTCGCAGCAT

TRINITY_DN17441   TTGGTTTCTTTGTCGAGACCTATAGAAAGTTGGAAAAAAACAAAAATAACAACAATCCATCGCCCTATACATCCAGTCGT
TRINITY_DN9159    --------------------------------------------------------------------------------
TRINITY_DN6833    TTGGTTTCTTTGTCGAGACCTATAGAAAGTTGGAAAAAAACAAAAATAACAACAATCCATCGCCCTATACATCCAGTCGT
Pvio_g6080        TTGGTTTCTTTGTCGAGACCTATAGAAAGTTGGAAAAAAACAAAAATAACAACAATCCATCGCCCTATACATCCAGTCGT

TRINITY_DN17441   GGCATTACATCTACACCAGGAGGTGGTAAGACTTTTACCACCAATGACAATCCAGATTCAATTGAAGTGCTAGAAATGGA
TRINITY_DN9159    --------------------------------------------------------------------------------
TRINITY_DN6833    GGCATTACATCTACACCAGGAGGTGGTAAGACTTTTACCACCAATGACAATCCAGATTCAATTGAAGTGCTAGAAATGGA
Pvio_g6080        GGCATTACATCTACACCAGGAGGTGGTAAGACTTTTACCACCAATGACAATCCAGATTCAATTGAAGTGCTAGAAATGGA

TRINITY_DN17441   ACCATGTCC-----------------------------------------------------------------------
TRINITY_DN9159    --------------------------------------------------------------------------------
TRINITY_DN6833    ACCATGTCCAATCTCCGAAGATGGAAATATTAGTACACCTGTGTTAACTGGTGCTGATGTAAATCCTAGAAATGAAGATT
Pvio_g6080        ACCATGTCCAATCTCCGAAGATGGAAATATTAGTACACCTGTGTTAACTGGTGCTGATGTAAATCCTAGAAATGAAGATT

TRINITY_DN17441   --------------------------------------------------------------------------------
TRINITY_DN9159    --------------------------------------------------------------------------------
TRINITY_DN6833    TAAATGCCGATTTAAGCGAAAACCATCAAAATGGATCAAGTGCTGTGTAAAATTATCTTTTCCAAAAAAACAAAACAAAA
Pvio_g6080        TAAATGCCGATTTAAGCGAAAACCATCAAAATGGATCAAGTGCTGTGTAAAATTATCTTTTCCAAAAAAACAAAACAAAA

TRINITY_DN17441   --------------------------------------------------------------------------------
TRINITY_DN9159    --------------------------------------------------------------------------------
TRINITY_DN6833    CATC----------------------------------------------------------------------------
Pvio_g6080        CATCTTTTTTTTAATTTTAAAATATTATAATAATAATTGTATTAATAGTAATAATAATAATGTCATTCATTTTCTTTTTTA

TRINITY_DN17441   ------------------------------------------------------------
TRINITY_DN9159    ------------------------------------------------------------
TRINITY_DN6833    ------------------------------------------------------------
Pvio_g6080        AAATAAATTATTATTGTATTATACTTCCCTTAATCAAAATTTGATTTTTTTAAATTCGTT
```

Fig. S7. Alignment of *Pvio* transcriptome and genome *car* hits

The *Pvio* genome and a *Pvio* transcriptome, assembled *de novo* (Schilde, Kin and Schaap, unpublished results) with the Trinity platform (Haas et al., 2013), were queried by tBLASTn, using *D. discoideum carA* as bait. The *Pvio* genome yielded a top hit (Pvio_g6080) at an E-value of $1.5x10^{-128}$, while the transcriptome yielded three hits at E-values varying from $1.2x10^{-145}$ to $3.9x10^{-60}$. The next hits with E-values of $6x10^{-11}$ for the genome and $1.3x10^{-12}$ for the transcriptome were Pvio_g6448, an ortholog of *Ddis lrlA* (lower E-values reflect higher sequence similarity). The top genome and top three transcriptome hits were aligned using ClustalOmega (Sievers and Higgins, 2014). The alignment above shows that the four sequences overlapped and were almost completely identical, i.e. part of the same *Pvio car* gene.

Table S1. Oligonucleotide primers used in this work

Name	Sequence
Pv-aca-51S2	TCCCCGCGGTTGTTGGCTGAAAGTGAGA
Pv-aca-31K	GGGGTACCTTCAAAGTCTGTGGCAGAACC
Pv-aca-P51K	GGGGTACCTAGACTCGAAGCTAGAGAAC
Pv-aca-P31B	CGGGATCCGTGGTGAATTGTGACTATAGGAC
Pv-aca-C31	GGACATTAGTGATGGACTCT
Pv-aca-C51	GGAATGGATTATGCATCGTTC
Pv-aca-31C	CCATCGATGAATCACATGGCTGGACATC
Pv-ACB-51K	GGGGTACCTTCCACCTTGGTCTCTGATG
Pv-ACB-31S	TCCCCGCGGTCTGCTGTTACCCCAGACAC
Pv-ACB-52K	GGGGTACCTTCATTCACCCCGTACTC
Pv-ACB-32	GGTCTTGATGTCCTTGCT
Pv-ACB-P51X	GCTCTAGAATCCAGCAGGATACACC
Pv-ACB-P31B	CGGGATCCCACTACTAGTTTGACGACT
Pv-ACB-C52	GGAGACGTCAAAATAGTAGCGA
Pv-ACB-C32	TCCGTTATCTTTGGGTTGAGG
Neo-3	GGGCCCCACCGTGGTTAATTAATTAACCCGGGAA
Pv-cAR-51K	GGGGTACCACACAAAAAAGAGAAGCAC
Pv-cAR-31C	CCATCGATGTTAGGGTTAAAATGGTAGAGG
Pv-cAR-52B	CGGGATCCTATCTTGGTAATTGGTGCTG
Pv-cAR-32X	GCTCTAGAGGATTTTATCTGTCTGCTGT
Pv-cAR-35K	GGGGTACCTTCACTGGTCTTTGCTGTCT
Pv-pdsA-51K	GGGGTACCATTAAATTCGCCCACCAC
Pv-pdsA-31	TCATCAAGACCACCAGCA
Pv-pdsA-52B	CGGGATCCTCAACAAGAAGCCATCA
Pv-pdsA-32	CTCATCTCTTTCCTCTCAC

SUPPLEMENTARY REFERENCES

Forbes, G., Chen, Z. H., Kin, K., Lawal, H. M., Schilde, C., Yamada, Y. and Schaap, P. (2019). Phylogeny-wide conservation and change in developmental expression, cell-type specificity and functional domains of the transcriptional regulators of social amoebas. *BMC Genomics* **20**, 890.

Gloeckner, G., Lawal, H. M., Felder, M., Singh, R., Singer, G., Weijer, C. J. and Schaap, P. (2016). The multicellularity genes of dictyostelid social amoebas. *Nature communications* **7**, 12085.

Haas, B. J., Papanicolaou, A., Yassour, M., Grabherr, M., Blood, P. D., Bowden, J., Couger, M. B., Eccles, D., Li, B., Lieber, M., et al. (2013). De novo transcript sequence reconstruction from RNA-seq using the Trinity platform for reference generation and analysis. *Nature protocols* **8**, 1494-1512.

Biology Open • Supplementary information

Kin, K., Forbes, G., Cassidy, A. and Schaap, P. (2018). Cell-type specific RNA-Seq reveals novel roles and regulatory programs for terminally differentiated Dictyostelium cells. *BMC Genomics* **19**, 764.

Parikh, A., Miranda, E. R., Katoh-Kurasawa, M., Fuller, D., Rot, G., Zagar, L., Curk, T., Sucgang, R., Chen, R., Zupan, B., et al. (2010). Conserved developmental transcriptomes in evolutionarily divergent species. *Genome Biol* **11**, R35.

Ronquist, F. and Huelsenbeck, J. P. (2003). MrBayes 3: Bayesian phylogenetic inference under mixed models. *Bioinformatics* **19**, 1572-1574.

Schultz, J., Milpetz, F., Bork, P. and Ponting, C. P. (1998). SMART, a simple modular architecture research tool: identification of signaling domains. *Proc. Natl. Acad. Sci. USA* **95**, 5857-5864.

Sievers, F. and Higgins, D. G. (2014). Clustal omega, accurate alignment of very large numbers of sequences. *Methods in molecular biology* **1079**, 105-116.

Singh, R., Schilde, C. and Schaap, P. (2016). A core phylogeny of Dictyostelia inferred from genomes representative of the eight major and minor taxonomic divisions of the group. *BMC Evol Biol* **16**, 251.

Trifinopoulos, J., Nguyen, L.-T., von Haeseler, A. and Minh, B. Q. (2016). W-IQ-TREE: a fast online phylogenetic tool for maximum likelihood analysis. *Nucleic Acids Research* **44**, W232-W235.

Biology Open • Supplementary information
